# New Insights into Non-Avian Dinosaur Reproduction and Their Evolutionary and Ecological Implications: Linking Fossil Evidence to Allometries of Extant Close Relatives

**DOI:** 10.1371/journal.pone.0072862

**Published:** 2013-08-21

**Authors:** Jan Werner, Eva Maria Griebeler

**Affiliations:** Department of Ecology, Zoological Institute, University of Mainz, Mainz, Germany; State Natural History Museum, Germany

## Abstract

It has been hypothesized that a high reproductive output contributes to the unique gigantism in large dinosaur taxa. In order to infer more information on dinosaur reproduction, we established allometries between body mass and different reproductive traits (egg mass, clutch mass, annual clutch mass) for extant phylogenetic brackets (birds, crocodiles and tortoises) of extinct non-avian dinosaurs. Allometries were applied to nine non-avian dinosaur taxa (theropods, hadrosaurs, and sauropodomorphs) for which fossil estimates on relevant traits are currently available. We found that the reproductive traits of most dinosaurs conformed to similar-sized or scaled-up extant reptiles or birds. The reproductive traits of theropods, which are considered more bird-like, were indeed consistent with birds, while the traits of sauropodomorphs conformed better to reptiles. Reproductive traits of hadrosaurs corresponded to both reptiles and birds. Excluding *Massospondylus* *carinatus* , all dinosaurs studied had an intermediary egg to body mass relationship to reptiles and birds. In contrast, dinosaur clutch masses fitted with either the masses predicted from allometries of birds (theropods) or to the masses of reptiles (all other taxa). Theropods studied had probably one clutch per year. For sauropodomorphs and hadrosaurs, more than one clutch per year was predicted. Contrary to current hypotheses, large dinosaurs did not have exceptionally high annual egg numbers (AEN). Independent of the extant model, the estimated dinosaur AEN did not exceed 850 eggs (75,000 kg sauropod) for any of the taxa studied. This estimated maximum is probably an overestimation due to unrealistic assumptions. According to most AEN estimations, the dinosaurs studied laid less than 200 eggs per year. Only some AEN estimates obtained for medium to large sized sauropods were higher (200-400 eggs). Our results provide new (testable) hypotheses, especially for reproductive traits that are insufficiently documented or lacking from the fossil record. This contributes to the understanding of their evolution.

## Introduction

The discovery of the gigantic sauropods and other large dinosaurs has stimulated scientists to understand the biology of dinosaurs. Several researchers [1–3] recently argued that the reproductive strategy of producing many small offspring contributed to the exceptional gigantism seen in the sauropods, a hypothesis introduced by Janis and Carrano [4] and recently corroborated by Werner and Griebeler [5].

In contrast to any living species, our information on dinosaurs and their reproduction is limited to fossils. Unfortunately, fossils do not allow for the complete reconstruction of an organisms’ traits (e.g. of the life history). Traits are often inaccurately preserved or simply absent from the fossil record (e.g. clutches can be incomplete and breeding frequency is simply not documented in the fossil record). This hampers our understanding of the reproductive strategies employed by dinosaurs.

Equations linking body mass to other traits derived from extant taxa are commonly used to estimate these traits for extinct species, including those dealing with reproduction (e.g. [6–8]). While the correlations between clutch/litter size or annual offspring number and body mass differ between different extant amniotic taxa [4,5,9,10], mass specific reproductive traits such as egg mass, clutch mass and annual reproductive mass (clutch mass × number of clutches per year) do significantly, positively correlate with body mass [9,11–17]. The relationships between traits (Y) and body mass (BM) follow a power function Y = *c* × BM^b^, where *c* is a normalization constant and *b* is an exponent. These so-called allometric (allometric because *b* usually differs from one) functions are usually log-log plotted, whereby the normalization constant *c* is the intercept and the exponent *b* is the slope of a straight line. The exponent *b* differs between different amniotic groups when egg mass is plotted against body mass [9,10]. Conversely, *b* is often very similar between different amniotic groups when clutch mass or annual reproductive mass is plotted against body mass [9,10,16]. While the slopes *b* are often statistically indistinguishable for annual clutch/litter mass, the normalization constants vary significantly among taxa [16,18]. These normalization constants are frequently similar among species/taxa with similar lifestyles [18]. We assume from these observations that analogous allometries on body mass and reproductive traits exist within close extant phylogenetic relatives of non-avian dinosaurs. We expect that these allometries are applicable to extinct non-avian dinosaurs as well.

To derive information on dinosaur reproduction, the procedure presented by Bryant and Russell [19] and Witmer [20] was employed, specifically a combination of phylogenetic inference and extrapolatory analysis. As an extant phylogenetic bracket (EPB, [20]) for non-avian dinosaurs, we chose phylogenetically close relatives with reproductive characteristics similar to those assumed for non-avian dinosaurs. Reproductive traits included, among others, ground breeding, and calcified, rigid-shelled eggs (for the distribution of egg types over the phylogeny of early amniotes, see Sander [21]). For the extant bird and reptile species which meet these specifications, we established allometries between body mass and different reproductive traits (egg mass, clutch mass, annual clutch mass). Allometries were then applied to nine non-avian dinosaur taxa for which information on body mass, egg mass and clutch mass is currently available from the fossil record. We expected, (i) reproductive trait estimates of non-avian dinosaurs, which are considered to be more “bird-like” in their reproductive mode (e.g. theropods [22–24]), to conform to those seen in extant birds. Similarly, those dinosaurs expressing traits probably more “reptile-like” in their reproductive mode (e.g. sauropods [24]) were expected to fit to those of extant reptiles.

Our established allometries between body mass and clutch mass might also provide further support for the hypothesis of Seymour [25], who argued that the buried clutch mass of large sauropods is limited due to physiological constrains imposed on the clutch. Assuming a sea turtle model, Seymour [26] showed that unfavorable respiratory gas pressures can occur inside large buried clutches. If clutches are too large, buried eggs do not receive enough oxygen through the soil [25]. Because of this physiological limitation of clutch mass, Seymour [25] suggested that fully buried sauropod eggs were distributed over several small clutches, each clutch containing not more than 13 eggs. Sander et al. [27] also hypothesized that the clutch mass of buried sauropod clutches is smaller than expected given their body mass, suggesting that these sauropods produced several clutches per year. According to these hypotheses, we expected (ii) the clutch masses of buried clutches of large dinosaurs, e.g. of the sauropods producing *Megaloolithusmammilare* eggs [27], to be smaller than those predicted from any extant species studied. Assuming that the annual clutch mass allometries derived from extant taxa are also valid for dinosaurs, (iii) we estimated annual breeding frequencies of dinosaurs from their fossil clutch mass. Finally, (iv) we estimated the total number of eggs laid per year for each dinosaur taxon, calculated from the annual clutch mass allometries of extant taxa using the respective fossil egg masses.

## Material and Methods

### Phylogenetic framework and EPB

For our analyses, we selected three extant taxa (birds N=217, crocodiles N=22 and tortoises N=20; Table S2), each of which is phylogenetically close to non-avian dinosaurs and/or has reproductive characteristics similar to non-avian dinosaurs. We chose the extant phylogenetic bracket of non-avian dinosaurs, i.e. birds and crocodiles, as the closest phylogenetic relatives of ancient dinosaurs [28–30]. Given that dinosaurs were most probably terrestrial and ground breeding [27,31,32], we also aimed to restrict our dataset to ground breeding and terrestrial bird species. We initially focused on avian orders presumed to have less derived reproductive characteristics (e.g. ground breeding and precocial). According to traditional taxonomy, these include Paleognathae with the orders Struthioniformes and Tinamiformes. Since the sample sizes obtained for Paleognathae were too small, we also included the orders Galliformes and Anseriformes in our analysis; both are phylogenetically closely related to Paleognathae and are ground breeding and precocial. For Struthioniformes, data from only seven species (two kiwi species and five other ratites) were available, with the two kiwi species (~ 1-3 kg, cave breeding) strongly differing from the other ratites (~ 20-90 kg, open breeding) in terms of body masses and reproductive strategies [33,34]. For Tinamiformes, we found no single species for which information on body mass and all studied reproductive traits was available. Our allometries were finally based on 60 galliforme, 150 anseriforme and 7 ratite species. The allometries obtained from these avian species are hereafter referred to as bird model.

For the crocodile model, we chose all extant crocodilian species (N = 22). Since crocodiles are non-terrestrial, we also included tortoises (N = 20) in our allometric analyses. Molecular data suggest that turtles are more closely related to archosaurs than to lepidosaurs [35–37] and, similar to crocodiles and birds, and tortoises have calcified, rigid-shelled eggs. For both the crocodile and tortoises model, all extant species for which information on reproductive traits and body mass was available were included in the model. For each of the three extant taxa, we established allometries linking reproductive traits of species to their body mass.

### Dinosaurs

We applied established allometries to all dinosaur taxa for which body mass estimates and assignments of fossil eggs or clutches to taxa are currently available (Table S1). These assignments only exist for four theropods ( *Troodon* *formosus* , *Oviraptor* *philoceratops* , *Citipati* *osmolskae* , and *Lourinhanosaurus* *antunesi* ), two hadrosaurs ( *Maiasaura* *peeblesorum* , lambeosaurine dinosaur), two sauropod oospecies ( *Megaloolithus* *patagonicus* , *Megaloolithusmammillare* ) and one prosauropod ( *Massospondylus* *carinatus* ). It should be noted that *Megaloolithussiruguei* is considered a junior synonym of *Megaloolithusmammilare* .

### Life-history traits

For all extant species, we gathered data on adult body mass (BM), egg mass (EM), clutch size (CS) and number of clutches per year (CY). When more than one trait value was found in the literature for the same species, the mean value was calculated (Table S2). For body mass estimates, data on females was preferentially used because mass is more strongly linked to reproductive traits in females than in males. In some cases, however, it was not possible to distinguish between male and female body masses because only the averages of both sexes were available or the sex was not denoted in the source or was unknown (especially for all dinosaurs). To maximize our sample size while minimizing any bias introduced by male body masses, we used female body masses wherever possible and otherwise averaged body masses.

For dinosaurs (for details, see Table S1, supporting information), we used the average species body masses for our estimations when eggs were assigned to a specific dinosaur species ( *Troodon* *formosus* , *Oviraptor* *philoceratops* , *Citipati* *osmolskae* *,* *Lourinhanosaurus* *antunesi* *,* *Maiasaura* *peeblesorum* *,* *Massospondylus* *carinatus* ). When eggs were assigned instead to a taxonomic group, we used averages of the specific taxonomic group ( *Megaloolithus* *patagonicus* , *Megaloolithusmammilare* , lambeosaurine dinosaur). Dinosaur egg masses were calculated from an egg’s volume, assuming an egg density of 1.13 g/cm^3^ (birds [38]:, mean egg density of the six bird orders from Table 3 in this reference; crocodiles [39]:, Table 3 in this reference). Egg volume was calculated from fossil egg dimensions (mean) using either the equation V = 0.51L* D^2^ (asymmetrical, bird-like, theropods) or V = 0.524L* D^2^ (ellipse/globular, crocodile-like, sauropodomorphs and hadrosaurs), where V is the egg volume, L the egg length and D the egg diameter [39,40].

**Table 3:** Reproductive characteristics of hadrosaurs as documented in the fossil record and estimated by the reptile (crocodiles, tortoises) and the bird model.

	**Fossil**			**Reptile model**				**Bird model**		
**Taxon**	**BM (kg)**	**EM (kg)**	**CS**	**CS^T^**	**CS^C^**	**AEN**	**CY**	**CS^B^**	**AEN**	**CY**
lambeosaurine	2390	4.737	22	3.5	9.9	10.3	0.5	23.2	25.7	1.2
lambeosaurine	3344	4.737	22	4.4	12.6	13.1	0.6	29.5	32.7	1.5
lambeosaurine	5057	4.737	22	6.0	16.9	17.7	0.8	39.7	44.1	2.0
*Maiasaura* *peeblesorum*	1500	1.023	16	11.6	32.9	34.0	2.1	77.1	84.8	5.3
*Maiasaura* *peeblesorum*	2556	1.023	16	16.9	48.2	50.1	3.1	112.8	124.7	7.8
*Maiasaura* *peeblesorum*	4079	1.023	16	23.7	67.3	70.2	4.4	157.6	174.9	10.9

BM: minimum, mean and maximum fossil body mass of a taxon taken from literature in kilograms; CS: mean clutch size observed in fossil record, with minimum and maximum fossil values given in square brackets or CS calculated from an allometric clutch mass (CM) model (CS^T^ = tortoise model, CS^C^ = crocodile model, CS^B^ = bird model) using the fossil egg mass (EM, for calculating of fossil egg mass see Material and Method), CS = CM divided by EM. AEN (annual egg number): total number of eggs laid per year, calculated from an allometric ACM (annual clutch mass) model using the fossil egg mass, AEN = ACM divided by EM. CY: number of clutches per year, calculated from an allometric ACM model using the fossil CS and estimated AEN, CY = AEN divided by CS. Minima and maxima are given in brackets. References for fossil data are given in Table S3 (supplementary). Equations for the reptile and the bird model are given in Table S6. Note: For the lambeosaurine and *Maiasaura* *peeblesorum* only one CS estimate was available.

Given that all studied extant birds and reptiles lay at least one clutch per year, an initial conservative estimate of the unknown annual breeding frequency was assumed to be one clutch per year for all non-avian dinosaurs. Clutch mass is egg mass multiplied by clutch size. Annual clutch mass is clutch mass multiplied by the number of clutches per year.

### Statistical analyses

#### Establishment of allometries for birds, crocodiles and tortoises

We began by separately analysing the relationship between body mass and reproductive traits for extant birds, crocodiles and tortoises. For each taxa and each of the three reproductive traits, we calculated regression slopes and normalization constants using ordinary least square regressions (OLS) on log-log-transformed data (Table S3).

In these regression analyses, we did not control for phylogenetic effects on reproductive traits. In general, phylogenetic comparative methods perform best when the phylogeny itself and branch lengths are correct [41–43]. However, to the best of our knowledge, no phylogenetic trees resolving to the species level are currently available for birds, crocodiles and tortoises. More notably, a phylogenetic tree containing all studied species with reliable branch lengths is not available. Furthermore, the purpose of taking phylogeny into account is to reduce the variance in the estimated regression or correlation coefficients. However, the estimates of regression coefficients are unbiased and independent of phylogeny; estimates of correlation coefficients are only slightly biased [44]. Because we were mainly interested in the mean coefficient values, and we obtained highly significant coefficients, controlling for phylogeny would not have improved our analyses.

Next, we tested the homogeneity of the regression lines obtained for the three taxonomic groups within each reproductive trait (Table S4, S5). Therein, the calculated regression slopes and the normalization constants of all taxonomic groups of one reproductive trait were compared (analyses of covariance ANCOVA, groups as categorical variable). When these overall analyses indicated that normalization constants and/or slopes of more than one taxonomic group differed for a reproductive trait, we used ANCOVA for an additional pairwise comparison. This aimed to identify potential differences or similarities in normalization constants and/or slopes within groups.

**Figure 1:**
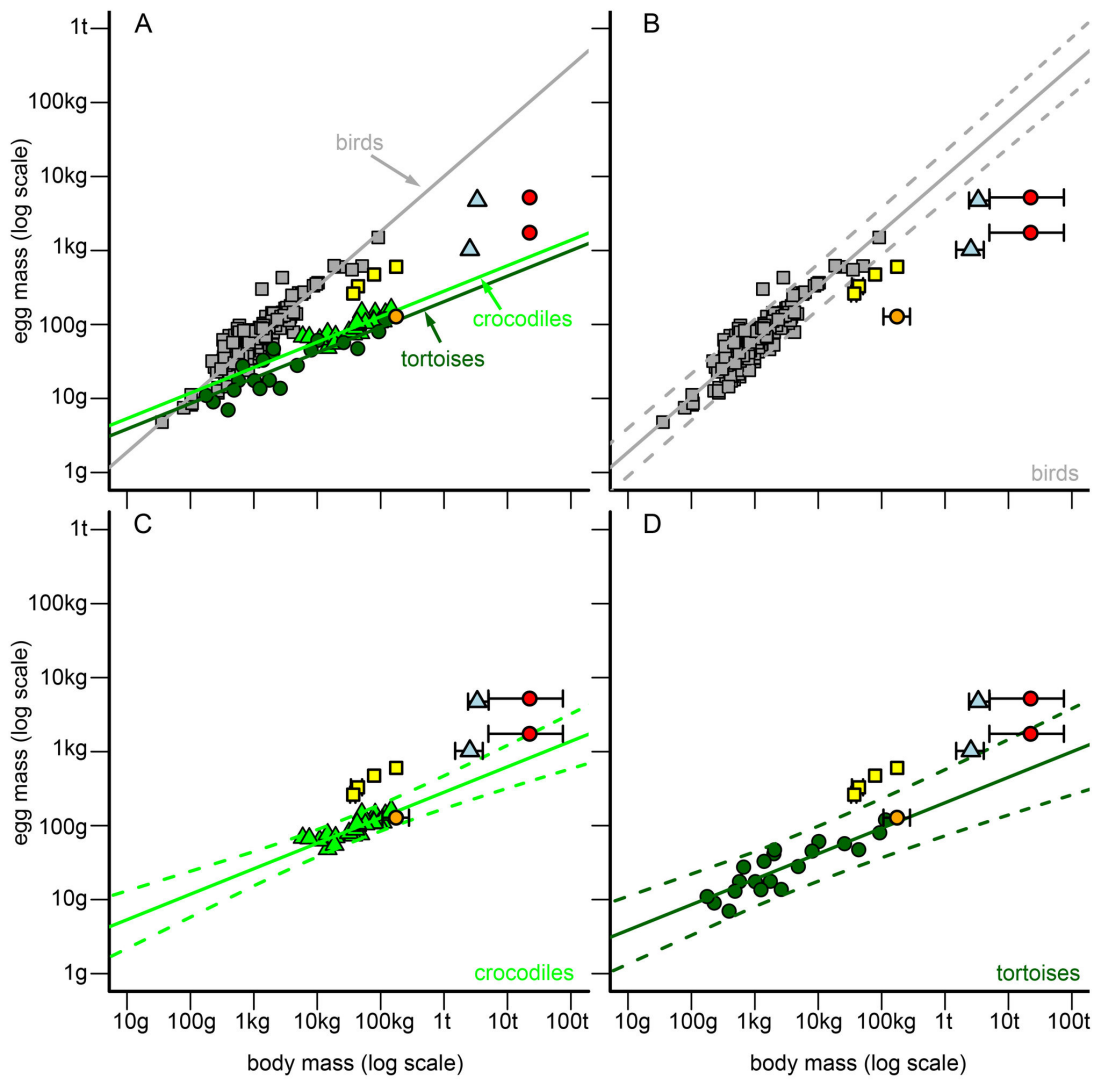
Allometries between body mass (BM) and egg mass (EM) of birds, crocodiles, and tortoises and their comparison to non-avian dinosaurs. (**A**) General comparison of dinosaur EMs to the EM allometry of birds (grey squares/line), crocodiles (green triangles/line) and tortoises (dark green circles/line). (**B**) Detailed comparison of dinosaur EMs to the EM allometry of birds. Grey continuous line = regression line of birds. Grey scattered lines = 95% prediction interval of the bird regression. Dinosaurs in the graphs = theropods (yellow squares, from left to right): *Troodon* *formosus* , *Oviraptor* *philoceratops* , *Citipati* *osmolskae* *,* *Lourinhanosaurus* *antunesi* ; hadrosaurs (light blue triangles, from bottom to top): *Maiasaura* *peeblesorum* , lambeosaurine dinosaur; sauropod oospecies (red circles, from bottom to top): *Megaloolithus* *patagonicus* , *Megaloolithusmammilare* ; prosauropod (orange circle): *Massospondylus* *carinatus* . Black error bars = possible value ranges for a non-avian dinosaur taxon (left/lower bar = minimum value derived from the fossil record, right/upper bar = maximum value). (**C**) Analogous to (B), but for crocodiles (green triangles/lines) and dinosaurs. (**D**) Analogous to (B), but for tortoises (dark green circles/lines) and dinosaurs.

**Figure 2:**
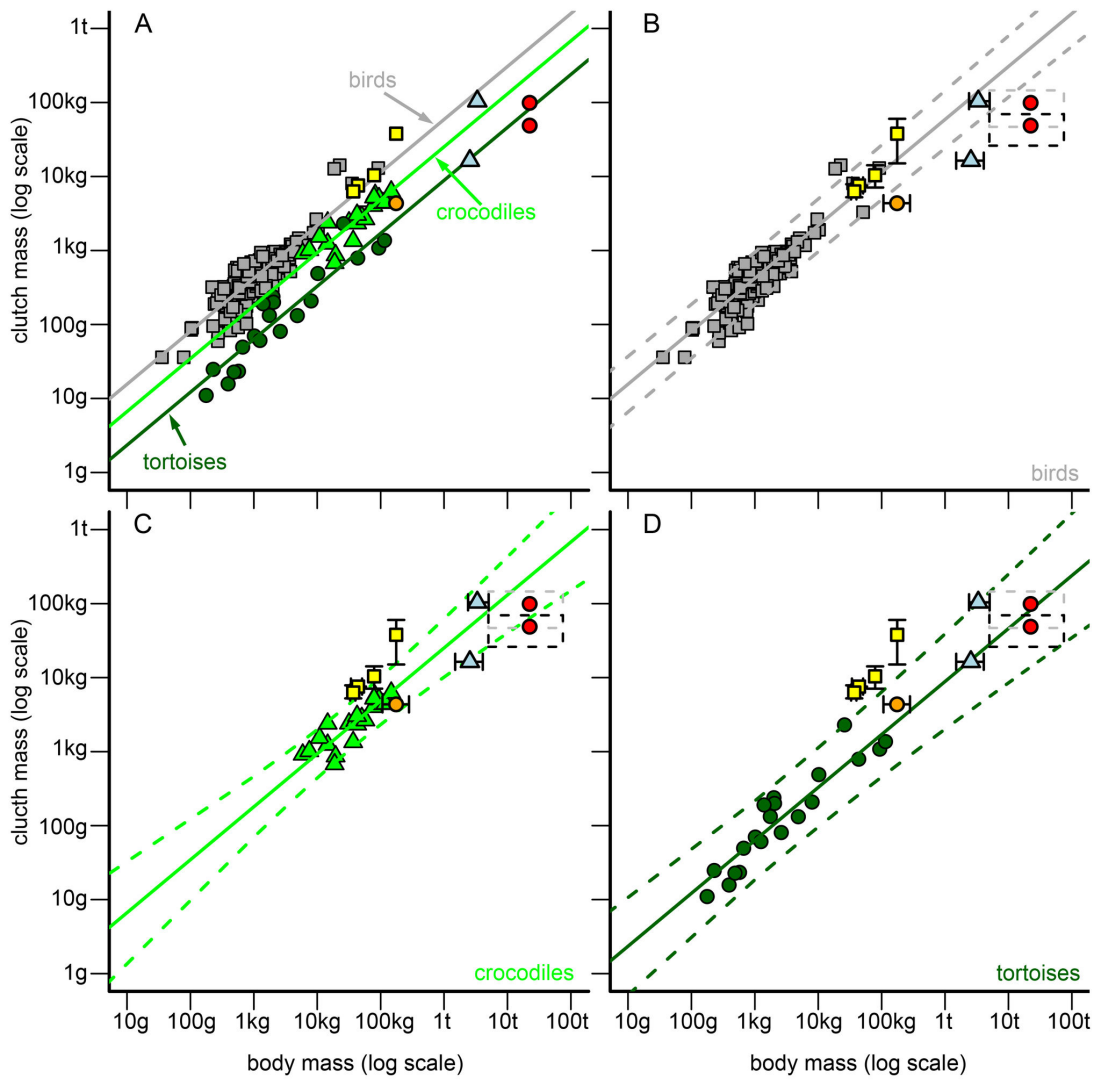
Allometries between body mass (BM) and clutch mass (CM) of birds, crocodiles, and tortoises and their comparison to non-avian dinosaurs. (**A**) General comparison of dinosaur CMs to the CM allometry of birds (grey squares/line), crocodiles (green triangles/line) and tortoises (dark green circles/line). (**B**) Detailed comparison of dinosaur CMs to the CM allometry of birds. Grey continuous line = regression line of birds. Grey scattered lines = 95% prediction interval of the bird regression. Dinosaurs in the graph = theropods (yellow squares, from left to right): *Troodon* *formosus* , *Oviraptor* *philoceratops* , *Citipati* *osmolskae* *,* *Lourinhanosaurus* *antunesi* ; hadrosaurs (light blue triangles, from bottom to top): *Maiasaura* *peeblesorum* , lambeosaurine dinosaur; sauropod oospecies (red circles, from bottom to top): *Megaloolithus* *patagonicus* , *Megaloolithusmammilare* ; prosauropod (orange circle): *Massospondylus* *carinatus* . Black error bars/scattered rectangles = possible value ranges for a non-avian dinosaur taxon (left/lower bar/edge = minimum value derived from the fossil record, right/upper bar/edge = maximum value). (**C**) Analogous to (B), but for crocodiles (green triangles/lines) and dinosaurs. (**D**) Analogous to (B), but for tortoises (dark green circles/lines) and dinosaurs.

**Figure 3:**
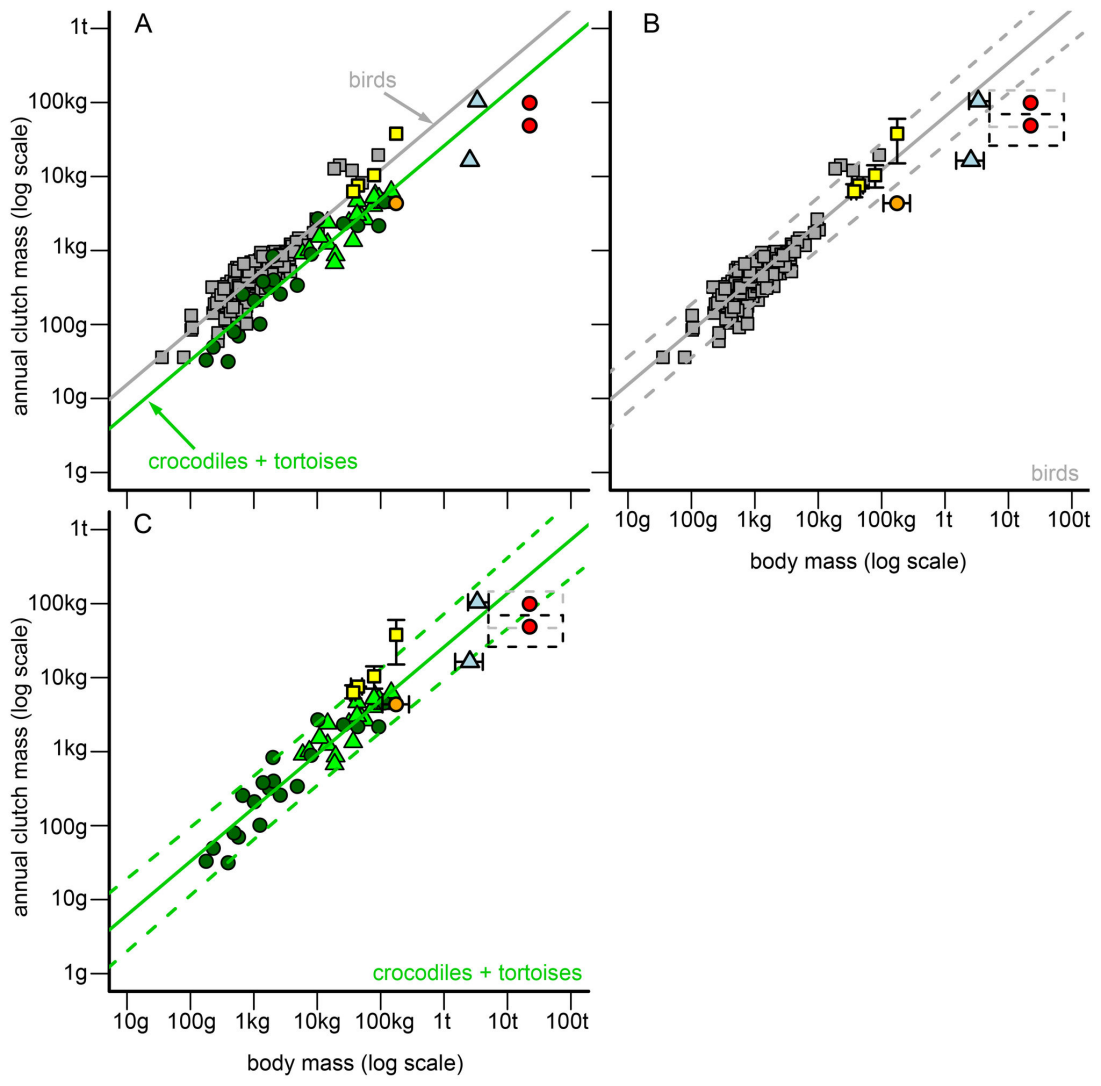
Allometries between body mass (BM) and clutch mass (ACM) of birds, crocodiles, and tortoises and their comparison to non-avian dinosaurs. (**A**) General comparison of dinosaur ACMs to the ACM allometry of birds (grey squares/line), crocodiles (green triangles/line) and tortoises (dark green circles/line). (**B**) Detailed comparison of dinosaur ACMs to the ACM allometry of birds. Grey continuous line = regression line of birds. Grey scattered lines = 95% prediction interval of the bird regression. Dinosaurs in the graph = theropods (yellow squares, from left to right): *Troodon* *formosus* , *Oviraptor* *philoceratops* , *Citipati* *osmolskae* *,* *Lourinhanosaurus* *antunesi* ; hadrosaurs (light blue triangles, from bottom to top): *Maiasaura* *peeblesorum* , lambeosaurine dinosaur; sauropod oospecies (red circles, from bottom to top): *Megaloolithus* *patagonicus* , *Megaloolithusmammilare* ; prosauropod (orange circle): *Massospondylus* *carinatus* . Black error bars/scattered rectangles = possible value ranges for a non-avian dinosaur taxon (left/lower bar/edge = minimum value derived from the fossil record, right/upper bar/edge = maximum value). (**C**) Analogous to (B), but for reptiles (crocodiles + tortoises, green triangles/lines) and dinosaurs.

The previous analyses could either reveal no statistical differences in regression slopes between at least two taxonomic groups or a significant difference between groups for a reproductive trait. If slopes were statistically homogeneous but intercepts differed between taxonomic groups, we calculated a new OLS regression function with a common regression slope for each group. We used this common regression slope (average value of taxa) as a fixed parameter in these regression models and only estimated the normalization constant for each group. If both slopes and intercepts of taxonomic groups were statistically homogeneous, we determined a common regression function (with the average slope and average intercept of taxa) for these taxa. If a slope of a single taxonomic group statistically differed from all other groups, the initially found OLS regression function was used as allometry for the respective taxon and reproductive trait (Table S6). This statistical procedure led to the development of three allometries/models for egg mass and clutch mass (birds, crocodiles, tortoises, Figures 1 and 2, Table S6) and two allometries for annual clutch mass (birds, reptiles = crocodiles + tortoises, see results, Figure 3, Table S6).

Finally, as a measure of variability in residuals and the deviation of single species from the expected average, we calculated 95% prediction intervals for each regression line (Figures 1-3).

#### Application of allometries to non-avian dinosaurs

Each of the trait pairs (body mass and reproductive trait) of non-avian dinosaurs were compared to the regression lines and to the respective 95% prediction intervals for the bird, the crocodile, and the tortoise model (Figures 1-3). Due to uncertainties in dinosaur body masses and clutch masses, we considered not only the mean fossil values but also their known variability (determined by reported minimum and maximum values taken from literature). For egg masses however, only the mean values were used. We assume that errors in egg mass are negligible in comparison to errors in the estimates of body mass and clutch size and mass.

#### Estimation of clutch sizes, annual egg numbers and clutches per year

Clutch size for non-avian dinosaurs was calculated from fossil egg mass and the clutch mass estimates from the regression lines derived for birds, crocodiles and tortoises. Analogously, the total number of eggs per year (AEN) for dinosaurs was calculated from annual clutch mass (ACM). Since the regression lines for crocodiles and tortoises did not differ statistically (see results), the ACM was estimated from the regression lines of birds and the common regression line for reptiles, and from the fossil egg mass. The number of clutches per year (CY) under the bird and reptile model was calculated from the respective annual egg numbers and from fossil clutch sizes.

#### Software used

The calculations of clutch sizes, number of eggs per year and clutches per year, estimated from the regression models, were done with Excel 2010. All other analyses were carried out in R (Version 2.14.1 [45]). For calculations of OLS regressions, common regression slopes and normalization constants, we used the “lm” function (basic) and the “gnls” function (“nlme” package) implemented in R. ANCOVAs were also conducted in R (“lm” function).

## Results

### Regression functions and estimation of dinosaur reproductive traits

Reproductive investment in terms of EM, CM, and ACM highly correlated with BM in birds, crocodiles and tortoises (Table S3). We found three different allometric models predicting EM from BM, with the models for crocodiles and tortoises only differing in their normalization constants (Tables S3-S6). For CM versus BM we also derived three different models; each model had different normalization constants (Tables S3-S6). For ACM versus BM we obtained only two different models (bird and reptile model, slopes and intercepts of the regression models of crocodiles and tortoises did not statistically differ) with different normalization constants (Tables S3-S6). All established models (Table S6) were used to compare the reproductive traits of dinosaurs documented in the fossil record with the respective reproductive traits seen in similar-sized or scaled-up extant species. The two models on ACM versus BM were applied to estimate AEN and CY for dinosaurs.

### Application of allometries to dinosaurs

#### Egg mass

Except for the prosauropod *M* *. carinatus* and the sauropod *M* *. patagonicus* , all dinosaur EMs fell outside the 95% prediction interval of all extant models (Figure 1, B–D). *Massospondylus* *carinatus* fitted very well to the crocodile and the tortoise model (Figure 1, C and D). The EM of *M* *. patagonicus* fitted the tortoise model, but only when we assume that its BM is equal to or higher than the mean BM of 22,399 kg, as given in literature (Figure 1D). None of the other dinosaur EMs fitted to the EM estimates of similar-sized or scaled-up birds or reptiles. Instead, they were intermediately located between these two models (Figure 1). In particular, the theropods ( *T* *. formosus* , *O* *. philoceratops* , *C* *. osmolskae* , and *L* *. antunesi* ) had EMs somewhat closer to those of birds than to reptiles, whereas the EMs of the hadrosaur *M* *. peeblesorum* and of the sauropod *M* *. patagonicus* were closer to those of reptiles (Figure 1). EMs of the lambeosaurine dinosaur (hadrosaur) and of the sauropod *M* *. mammilare* ranged between the predicted EM in the bird and the two reptile models (Figure 1).

#### Clutch mass

All fossil dinosaur CMs fell within the 95% prediction interval of at least one of the extant models (Figure 2, B–D). All non-theropod CMs matched at least one of the two reptile models (crocodiles, tortoises, Figure 2, C and D), while all theropod CMs matched the bird model (Figure 2B). However, the extant model best suited for the CM of a specific dinosaur differed between dinosaur taxa. Theropod CMs and the CM of the lambeosaurine dinosaur clearly conformed best to the bird model (Figure 2B), but the lambeosaurine dinosaur was in the 95% prediction interval of the crocodile model, too (Figure 2C). Sauropod CMs and the CM of the hadrosaur *M* *. peeblesorum* matched the tortoise model best, but were still realistic under the crocodile model (Figure 2, C and D). The CM of the prosauropod *M* *. carinatus* corresponded well to both the crocodile and the tortoise model (Figure 2, C and D). The CM of the sauropod *M* *. mammilare* fell within the 95% prediction interval of the crocodile model (Figure 2C). The CM of the sauropod *M* *. patagonicus* and of the hadrosaur *M* *. peeblesorum* also conform to the crocodile model (Figure 2C) when assuming a lower BM for both taxa ( *M* *. patagonicus* : 5,000-10,000 kg, *M* *. peeblesorum* : minimum reported BM of 1,500 kg), or in the case of *M* *. patagonicus* when the maximum CS reported in literature was used for predicting CM (40 eggs).

#### Annual clutch mass

When assuming one clutch per year, all estimated ACMs of non-avian dinosaurs fell within the 95% prediction interval of at least one of the extant models, with the exception of the sauropod *M* *. patagonicus* and the hadrosaur *M* *. peeblesorum* (Figure 3). All ACMs of theropods conformed well to the bird model (Figure 3B). In contrast, with the exception of the lambeosaurine dinosaur, none of the non-theropod dinosaur ACMs coincided with the bird model when assuming one clutch per year (Figure 3B). The ACM of the lambeosaurine dinosaur was intermediary when referring to the mass expected under the bird model and the reptile model (Figure 3). For the sauropod *M* *. patagonicus* and the hadrosaur *M* *. peeblesorum* , the ACMs derived from the assumption of one clutch per year coincided with the reptile model only when assuming body masses lower than the mean body masses derived from literature (Figure 3C). Combining *M* *. patagonicus* EMs and sauropod BMs with the maximum CS of 40 eggs reported in literature also leads to an ACM that conforms to the reptile model. ACMs of very large sauropods (75,000 kg and more) conformed neither to the bird nor to the reptile model when assuming the CS and EM reported in literature (for *M* *. patagonicus* or *M* *. mammilare* ) and only one clutch per year (Figure 3)*.*

### Estimates of annual egg numbers and clutches per year from allometries

Independent of the extant model used, the estimated dinosaur AEN did not exceed 850 eggs (75,000 kg sauropod) for any of the taxa studied. According to most estimations, dinosaurs lay less than 200 eggs per year and only some estimates obtained for medium to large sized sauropods were higher (Table 1). Assuming the bird model, for example, the AENs of theropods were comparable to fossil clutch sizes. This suggests that theropods had one clutch per year (Table 2). By contrast, hadrosaurs and sauropodomorphs probably had more than one clutch per year. This is supported by AEN estimates of nearly all BM and CM combinations (minimum, maximum and average values for BM and CM are considered). For all hadrosaurs and sauropodomorphs, these AEN estimates exceeded the egg number of fossil clutches, independent of the extant model assumed. The AENs of the lambeosaurine dinosaur calculated under the reptile and bird model indicated that this taxon probably had one or a maximum of two clutches per year. The *M* *. peeblesorum* had at least two clutches per year under the reptile model, and up to 11 clutches under the bird model (Table 3). Depending on the extant models applied for *M* *. carinatus* this taxon might have had one up to two (reptile model) or three up to six (bird model) clutches per year (Table 1). Small / young sauropods (BM ~ 5,000 kg), likely producers of oospecies *M* *. mammilare* or *M* *. patagonicus* eggs, might have had one (reptile model) up to two (bird model) or two (reptile model) up to four (bird model) clutches per year (Table 1). Medium sized sauropods (BM ~ 22,399 kg) might have laid two or three ( *M* *. mammilare* , reptile model), but up to six ( *M* *. mammilare* , bird model) clutches per year, depending on the model employed. For *M* *. patagonicus* , the number of clutches per year ranged from five (reptile model) to a maximum of 13 ( *M* *. patagonicus* , bird model). The AEN estimate for very large sauropods (BM ~ 75,000 kg) was also variable depending on the species and model used. Estimates range from six ( *M* *. mammilare* , reptile model) up to 15 ( *M* *. mammilare* , bird model) or even 13 ( *M* *. patagonicus* , reptile model) up to 30 ( *M* *. patagonicus* , bird model) clutches per year (Table 1).

**Table 1:** Reproductive characteristics of sauropodomorphs as documented in the fossil record and estimated by the reptile (crocodiles, tortoises) and the bird model.

	**Fossil**			**Reptile model**				**Bird model**		
**Taxon**	**BM (kg)**	**EM (kg)**	**CS**	**CS^T^**	**CS^C^**	**AEN**	**CY**	**CS^B^**	**AEN**	**CY**
*Massospondylus* *carinatus*	107	0.128	34	14.0	39.7	40.2	1.2	93.1	100.0	2.9
*Massospondylus* *carinatus*	175	0.128	34	19.9	56.5	57.3	1.7	132.4	142.9	4.2
*Massospondylus* *carinatus*	280	0.128	34	27.8	79.1	80.6	2.4	185.3	200.8	5.9
*Megaloolithusmammilare*	5000	5.211	19 [9, 28]	5.4	15.3	16.0	0.8 [0.6, 1.8]	35.8	39.8	2.1 [1.4, 4.4]
*Megaloolithusmammilare*	22399	5.211	19 [9, 28]	15.7	44.7	47.3	2.5 [1.7, 5.3]	104.7	118.0	6.2 [4.2, 13.1]
*Megaloolithusmammilare*	75000	5.211	19 [9, 28]	37.3	106.1	113.6	6.0 [4.1, 12.6]	248.6	283.1	14.9 [10.1, 31.5]
*Megaloolithus* *patagonicus*	5000	1.741	28 [15, 40]	16.1	45.7	47.8	1.7 [1.2, 3.2]	107.2	119.1	4.3 [3.0, 7.9]
*Megaloolithus* *patagonicus*	22399	1.741	28 [15, 40]	47.1	133.8	141.7	5.1 [3.5, 9.4]	313.4	353.0	12.6 [8.8, 23.5]
*Megaloolithus* *patagonicus*	75000	1.741	28 [15, 40]	111.8	317.6	340.1	12.1 [8.5, 22.7]	744.0	847.3	30.3 [21.2, 56.5]

BM: minimum, mean and maximum fossil body mass of a taxon taken from literature in kilograms; CS: mean clutch size observed in fossil record, with minimum and maximum fossil values given in square brackets or CS calculated from an allometric clutch mass (CM) model (CS^T^ = tortoise model, CS^C^ = crocodile model, CS^B^ = bird model) using the fossil egg mass (EM, for calculating of fossil egg mass see Material and Method), CS = CM divided by EM. AEN (annual egg number): total number of eggs laid per year, calculated from an allometric ACM (annual clutch mass) model using the fossil egg mass, AEN = ACM divided by EM. CY: number of clutches per year, calculated from an allometric ACM model using the fossil CS and estimated AEN, CY = AEN divided by CS. Minima and maxima are given in brackets. References for fossil data are given in Table S3. Equations for the reptile and the bird model are given in Table S6. Note: For *Massospondylus* *carinatus* only one CS estimate was available.

**Table 2:** Reproductive characteristics of theropods as documented in the fossil record and estimated by the reptile (crocodiles, tortoises) and the bird model.

	**Fossil**			**Reptile model**				**Bird model**		
**Taxon**	**BM (kg)**	**EM (kg)**	**CS**	**CS^T^**	**CS^C^**	**AEN**	**CY**	**CS^B^**	**AEN**	**CY**
*Troodon* *formosus*	34	0.329	23 [22, 24]	2.4	6.8	6.8	0.3 [0.3, 0.3]	15.9	17.0	0.7 [0.7, 0.8]
*Troodon* *formosus*	44	0.329	23 [22, 24]	2.9	8.2	8.2	0.4 [0.3, 0.4]	19.2	20.4	0.9 [0.9, 0.9]
*Troodon* *formosus*	51	0.329	23 [22, 24]	3.2	9.1	9.1	0.4 [0.4, 0.4]	21.3	22.8	1.0 [0.9, 1.0]
*Oviraptor* *philoceratops*	33	0.262	24 [20, 30]	2.9	8.4	8.4	0.3 [0.3, 0.4]	19.6	20.8	0.9 [0.7, 1.0]
*Oviraptor* *philoceratops*	37	0.262	24 [20, 30]	3.2	9.1	9.1	0.4 [0.3, 0.5]	21.3	22.6	0.9 [0.8, 1.1]
*Oviraptor* *philoceratops*	40	0.262	24 [20, 30]	3.4	9.6	9.6	0.4 [0.3, 0.5]	22.5	24.0	1.0 [0.8, 1.2]
*Citipati* *osmolskae*	79	0.473	22 [15, 30]	3.0	8.7	8.7	0.4 [0.3, 0.6]	20.3	21.7	1.0 [0.7, 1.4]
*Lourinhanosaurus* *atunesis*	176	0.602	63 [25, 100]	4.2	12.1	12.2	0.2 [0.1, 0.5]	28.3	30.5	0.5 [0.3, 1.2]

BM: minimum, mean and maximum fossil body mass of a taxon taken from literature in kilograms; CS: mean clutch size observed in fossil record, with minimum and maximum fossil values given in square brackets or CS calculated from an allometric clutch mass (CM) model (CS^T^ = tortoise model, CS^C^ = crocodile model, CS^B^ = bird model) using the fossil egg mass (EM, for calculating of fossil egg mass see Material and Method), CS = CM divided by EM. AEN (annual egg number): total number of eggs laid per year, calculated from an allometric ACM (annual clutch mass) model using the fossil egg mass, AEN = ACM divided by EM. CY: number of clutches per year, calculated from an allometric ACM model using the fossil CS and estimated AEN, CY = AEN divided by CS. Minima and maxima are given in brackets. References for fossil data are given in Table S3 (supplementary). Equations for the reptile and the bird model are given in Table S6. Note: For *Citipati* *osmolskae* and *Lourinhanosaurus* *antunesi* only one BM estimate was available.

## Discussion

### The allometries of body mass and reproductive investment in extant amniotes

Our results corroborate that body mass and reproductive investment (in terms of egg mass, clutch mass or annual clutch mass) are highly correlated in extant reptiles and birds [9,11–17]. In amniotes, the relative reproductive investment generally declines with body mass, whereas the absolute reproductive investment increases ( [16,46], Figures 1-3, Table S3). Our analysis provides additional evidence [9,15] that the egg mass of large birds is higher compared to similar-sized reptiles (Figure 1). In contrast, large reptiles have a larger number of eggs per clutch and/or per year than similar-sized birds [9,15]. This results in less distinction between clutch masses/annual clutch masses of large birds and reptiles than in egg masses (Figures 1, 2 and 3).

### Reproductive investment in dinosaurs

In summary, our results revealed four important insights into dinosaur reproductive biology. First, corroborating our hypothesis (i), the reproductive traits of dinosaurs that are considered to be more bird-like (theropods) did indeed coincide with reproductive traits of birds. Similarly, those traits of dinosaurs that were probably more reptile-like (prosauropods, sauropods) coincided with those of reptiles. Second, although the size difference between a dinosaur egg and the egg-laying female is very impressive, for all dinosaurs studied the egg to body mass relationship was similar to similar-sized or scaled-up extant reptiles (in *M* *. carinatus* ) or even higher (in all other dinosaurs). However, it was lower than in similar-sized or scaled-up birds. Third, contrary to our hypothesis (ii) clutch masses of all dinosaurs and even of sauropods matched at least one of the extant models. We thus did not find any evidence that sauropods clutch sizes are small in comparison to their body mass. This in turn questions the idea that a physiological limitation imposed on the clutch [25] leads to the “small” clutch size of fully buried sauropod clutches. Under such a limitation, the predicted CM to BM relationship would be too high in large dinosaurs, regardless of the extant model used. Fourth, annual clutch mass estimates (iii) suggest that theropods had only one clutch per year, whereas all other studied dinosaurs had probably several clutches per year (except for the lambeosaurine hadrosaur, for which one clutch per year is also realistic). This is especially true for the large sauropods. However, contrary to our expectation (iv), most of the dinosaurs studied probably laid no more than 200 eggs per year (Tables 1, 2 and 3). Even large sauropods (75,000 kg) probably had less than 400 eggs per year (Table 1), which is a smaller annual egg number than extant sea turtles (up to 513 eggs [47]).

#### Egg mass

Our results suggest that the egg masses of most dinosaurs match neither the egg masses of similar-sized or scaled-up birds nor those of reptiles, but were in fact in-between (Figure 1). This could reflect the reproductive strategy differences of most dinosaurs compared to the reproductive strategy seen in extant birds or reptiles [1] and suggests that their reproductive strategy was intermediary [24]. The great variability in egg mass to body mass relations found in dinosaurs (Figure 1) could indicate that different reproductive strategies existed in dinosaurs. The suggested variability in reproduction strategies is corroborated by the variability seen in dinosaur egg shapes and eggshell structures [31,32,48,49]. As observed in extant reptiles and birds, dinosaur egg mass (EM) increased significantly with body mass (BM; EM = 0.090* BM^0.311^ p= 0.031; r = 0.680 (Pearson’s correlation coefficient); N = 9, all dinosaur taxa studied). According to the assumption that the reptile reproductive model is plesiomorphic and the bird model is phylogenetically derived, none of the studied dinosaurs with egg masses close to the reptile model belong to the theropods (Figure 1). Furthermore, the egg mass of the most basal sauropodomorph ( *M* *. carinatus* ) matched both the crocodile and tortoise model well. Thus our results corroborate our hypothesis (i).

#### Clutch mass/size

In contrast to the egg masses, all dinosaur (mean) clutch masses matched the masses of similar-sized or scaled-up birds or reptiles.

Theropods. As expected under our initial hypothesis, the bird model was the best model for theropods. Fossils indicate that at least some avian reproductive characteristics, such as adult brooding [22,50–52], asymmetrical eggs [22,50,53,54], unornamented eggshell surface and complex eggshell ultrastructure, existed in non-avian theropods [55]. Thus, our results provide further evidence of a bird-like reproduction mode in theropods. Furthermore, our results on theropods suggest accurate body mass and egg mass estimates and the completeness of fossil clutches. The stronger deviation of *L* *. antunesi* from the bird model could possibly be explained by a higher inaccuracy in the estimates of its body mass (a not fully grown sub-adult individual is the holotype of this taxon, Mateus et al. [56]) and fossil clutch size (eggs of the clutch could come from different females [57]) than in the other theropods.

Varricchio et al. [23] assumed that some theropods received/ provided paternal care, because clutch-associated adults lack the maternal and reproductively associated histological feature common to extant archosaurs, including the medullary bone. Furthermore, theropods have relatively large clutch volumes. However, our analyses revealed no large clutch masses relative to body masses for theropods when compared to the studied extant birds showing bipaternal or maternal care. Thus, our data provide no evidence for the postulation presented in Varricchio et al. [23], that the theropods “sitting” on eggs were really males. The discrepancy in the results could have been caused by different sample compositions. We focused only on precocial birds in our analyses and used female body masses, as far as possible. We did not take into consideration the different parental care strategies of species. In contrast, Varricchio et al. [23] mixed different development modes of birds and used body mass averages without accounting for differences between sexes, but did allow for different parental care strategies. However, a recent study [58] corroborates our conclusion, showing that the development mode is a better predictor of the parental care strategy than clutch mass.

Sauropodomorpha. Contrary to our initial hypothesis (ii) clutch masses of sauropods were consistent with an extant species model, the tortoise model (Figure 2D). Several authors have argued that the clutch sizes of buried clutches in sauropods are bounded by physiological constrains [25,27,59], resulting in lower clutch mass to body mass relations compared to smaller taxa. Our analysis showed that the mean clutch masses for all studied dinosaurs matched the 95% prediction interval of at least one of the extant species models (birds, crocodiles or tortoises). Hence, they could be still consistent with the extant variability. For the two analyzed sauropods, the largest dinosaurs in our dataset, body mass and clutch size (particularly for *M* *. mammilare* ) [27,49, but see 60] is uncertain. However, even when assuming large errors in the body mass and clutch mass/size estimates for these two sauropods (Figure 2, scattered rectangles), the clutch mass to body mass relations did not conflict with those seen in scaled-up recent taxa (Figure 2D, the rectangles are completely located within the 95% prediction interval of the tortoise model). Additionally, the clutch mass of the prosauropod *M* *. carinatus* is also well described by the tortoise model (Figure 2D). All these observations suggest that the tortoise model might be appropriate for sauropodomorphs in general. Thus, our results provided no evidence that the “small” clutch sizes of *M* *. mammilare* are caused by physiological limits imposed on the clutch [25,27,59]. We think that the use of a sea turtle model, as Seymour [25] did, to determine physiological limits on a large buried sauropod clutch is problematic. Sea turtles bury their clutches much deeper than most other reptiles [61]. In crocodilian clutches, for example, the respiratory gas pressure is closer to the atmospheric level than in sea turtle clutches [61]. In a buried clutch of the turtle species *Chelodina* *expansa* the respiratory gas pressure is also similar to the atmospheric pressure [62]. Thus, oxygen availability plays a stronger role in sea turtle clutch size than in other egg-burying reptiles, and is presumably not such a limiting factor in sauropods.

Hadrosaurs. The applicability of allometric models for clutch mass differed between the two hadrosaurs. For the lambeosaurine hadrosaur, the bird model was best, but the crocodile model was also applicable. For *M* *. peeblesorum* , the tortoise model was best; the crocodile model was also applicable, but only when assuming the lowest body mass estimates for that species. This discrepancy could indicate that reproduction strategies differed in hadrosaurs, as already suggested by Horner [63]. However, our results could be biased by an incomplete *M* *. peeblesorum* fossil clutch count. This would lead to a low assumed clutch mass. Horner [63] noted that counting individual eggs in a *M* *. peeblesorum* clutch was very difficult and for this reason assumed that one clutch consisted of at least 16 eggs.

#### Annual clutch mass/clutch per year/annual egg number

A reliable estimate of the number of clutches per year and the annual egg number for dinosaurs is uncertain because of the high variability observed in traits of extant species. Furthermore, there is a high inaccuracy in body mass and clutch mass estimates of dinosaurs, making it difficult to completely rule out any of the extant models for dinosaur taxa. Irrespective of all these limitations, we are able to provide qualitative estimates for the number of clutches per year and the annual egg number laid by dinosaurs. By using further information from the fossil record, we were able to identify the most likely extant model for a taxon.

Theropods. Fossil egg masses and clutch masses of theropods are consistent with the bird model, which suggests a general applicability of allometries on reproductive traits of birds to theropods. Assuming one clutch per year for theropods, the theropod ACMs match the bird model (Figure 3B). Furthermore, the low annual breeding frequency of theropods might provide further evidence for parental care in this taxon [23].

Sauropodomorpha. ACMs of sauropodomorphs were lower than all “average” extant species studied when assuming the studied sauropodomorphs (Figure 3) to have one clutch per year and mean values for BM and CM/CS. However, with the exception of *M* *. patagonicus* , ACMs still fall within the 95% prediction interval of the reptile model (Figure 3C). Due to this observation and the finding that sauropods/sauropodomorphs were more “reptile-like” in their reproductive mode [24], we think that the reptile model is more appropriate for Sauropodomorpha than the bird model. The bird model provided high numbers of clutches per year, which could be flawed. These high estimates could be the result of incomplete fossil clutches (estimated clutch size is too small) or an assumed body mass too large for the producer. It is also possible that the bird model is not applicable for sauropods in general because of the more “reptile-like” reproduction strategy in sauropods.

Under the reptile model, an ”average” *M* *. carinatus* could have had one or a maximum of two clutches, and laid between 40 and 81 eggs per year. Our estimates of the number of clutches per year for sauropods are more imprecise than those for the theropods and prosauropod. The two studied oospecies were assigned to the taxon *Titanosauria*, which covers a wide range of body masses for the potential egg producer. However, even if the body mass of a fully grown adult would be known, a wide range of body masses for the egg producer is still possible because sauropods probably became sexually mature well before they were fully-grown [64,65]. Irrespective of all these uncertainties, the reptile model revealed reliable estimates of the number of eggs and clutches per year for Sauropodomorpha. A sauropod weighing 75,000 kg could have laid the eggs of the *M* *. patagonicus* oospecies and is predicted to have had twelve clutches per year. This would result in an annual egg number of 340. However, the small eggs of the *M* *. patagonicus* oospecies imply that they were likely produced by a small to medium sized sauropod. Based on the smaller clutch size found in *M* *. mammilare* compared to *M* *. patagonicus* , Sander et al. [27] argued that *M* *. mammilare* had several clutches per year, whereas *M* *. patagonicus* had only one clutch per year. Contrary to these authors, our results indicate that both sauropods had multiple clutches per year, assuming that the egg laying individuals were not very small sauropods (BM < 5,000 kg, Table 1). Based on the reptile model, we suggest that *M* *. patagonicus* had between two and five clutches per year resulting in an annual egg production between 48 and 142, whereas *M* *. mammilare* could have laid one up to six clutches per year resulting in around 16 but up to 114 eggs per year.

Altogether, our results imply that sauropods probably had several clutches per year, resulting in more than one hundred eggs per year. However, sauropod clutch and egg numbers probably did not exceed the numbers found in some recent reptile species (e.g. sea turtles [66]). Nevertheless, the high annual egg numbers estimated for Sauropodomorpha in comparison to other non-avian dinosaurs, recent birds or mammals could indicate a high predation rate of hatchlings and little parental care in this dinosaurian lineage.

Hadrosaurs. We could not clearly identify the most likely extant model for the ACM of hadrosaurs. The ACM to BM relation of the lambeosaurine hadrosaur was intermediary to both the bird and reptile model; for *M* *. peeblesorum* its relation was lower than observed in any extant model. Nevertheless, our results suggest that hadrosaurs probably had several clutches per year. For the lambeosaurine hadrosaur, one (reptile model) but up to two clutches (bird model) per year is estimated (Table 3). *Maiasaura* *peeblesorum* probably had more clutches per year than the lambeosaurine hadrosaur. The reptile model estimates two, up to four clutches per year and the bird model five up to eleven (Table 3). However, as discussed before, the assumed clutch size and mass of *M* *. peeblesorum* could be too low. The resulting number of clutches per year estimated could therefore be much closer to those of the lambeosaurine hadrosaur. In any case, the annual egg numbers of hadrosaurs studied were less than 200 (Table 3).

### Ecological implications

Why should some dinosaurs have several clutches per year and others not? As seen in extant species, they could have lived in different environments, each favoring different reproductive strategies. Producing several small clutches within a breeding season could reflect a bet-hedging strategy [67–69]. If few of many clutches are lost to predation or other unfavorable environmental conditions cause considerable egg mortality, the eggs/hatchlings from other clutches may survive by chance. Such a strategy is favorable in environments with a long breeding season and when the time intervals between clutches are relatively long. This is also true in environments with a short breeding season, when clutches are laid more or less simultaneously. Dinosaurs which had one clutch per year could have lived in environments with a short breeding season. Under such environmental conditions putting all eggs into a single clutch/reproductive event (producing all eggs in a specific time period) is the only option, even at high rates of egg mortality, because the length of the breeding season limits reproduction [70]. Irrespective of the length of the breeding season, one clutch per year can be sufficient if offspring mortality due to environmental conditions is low.

### Evolutionary implications

Since birds are dinosaurs, there must have been an evolutionary shift in the reproductive mode from the basal reptilian/non-avian dinosaur mode to that currently observed in birds. This shift might be observable in the studied dinosaurs. As expected, all studied reproductive traits of Sauropodomorpha were more reptile-like, whereas traits of studied theropods conform well to those of recent birds. Furthermore, it is likely that within the dinosaur lineage (including birds), an increase in egg size was linked to a decrease in egg numbers per clutch/year and vice versa. The prosauropod had many small eggs for its body mass. The two sauropods had in fact larger eggs than the prosauropod, but they still had many eggs in comparison to other dinosaurs because of their large size (Figure 1, Tables 1, 2 and 3). The four theropods had larger but fewer eggs than the sauropodomorphs (Figure 1, Tables 1 and 2), whereas recent birds have the largest eggs in comparison to their body mass (Figure 1) and also the fewest egg numbers (2.2-4.5 eggs per clutch, geometric mean of 5290 bird species [33]). This evolutionary change in egg size and number observed in the dinosaurian clade probably coincides with other changes in life history traits. High mortality during the egg and juvenile phase could have led to the evolution of and selection for parental care; in birds, this would have sustained viable populations over evolutionary time. A similar shift in life history traits might have also occurred in the hadrosaurian lineage (Ornithischia). Interestingly, other researchers have also assumed extended parental care for species of this lineage based on fossil clutches with “altricial hatchlings” (e.g. *Maiasaura* *peeblesorum* [71,72], but see 48,73). However, our results imply that extended parental care was more likely in the studied lambeosaurine hadrosaur than in *M* *. peeblesorum* . This is due to the increased size observed in eggs and estimated fewer egg/clutch numbers in the former taxon.

## Conclusion

From our study we conclude i) that allometric regression functions are a suitable approach to describe the relation between body mass and the studied reproductive traits in birds or reptiles. It is ii) appropriate to transfer these established allometries to specific taxa of extinct non-avian dinosaurs. Although we found a high variability in reproductive traits around the (average) allometric regression lines in extant species, we think that the results provide new testable hypotheses about dinosaur reproduction, its evolution and their ecological implications, especially for reproductive traits that are insufficiently documented or lacking in the fossil record.

## Supporting Information

Table S1 **Average body mass and reproductive characteristics of dinosaurs as documented in the fossil record.** The *Megaloolithus* *patagonicus* oospecies is assigned to a titanosaurian sauropod based on embryonic remains in the eggs, and the *Megaloolithussiruguei* /*mammilare* egg type (with a highly porous shell) is commonly assigned to titanosaurian sauropod dinosaurs, because titanosaur bones had been found in the same horizon or formation as the eggs. It should be noted that *Megaloolithussiruguei* is considered as a junior synonym of *Megaloolithusmammillare* . Megaloolithus eggs have also been assigned to titanosaurs because of the find of a hatchling in a nest of Megaloolitgus eggs from India. Although taxonomic identification of the eggs and their producers is problematic in sauropods, species with a mass of at least 5000 kg were assigned to both *Megaloolithus* oospecies. BM = body mass. ES = egg size, expressed in length (L) and diameter (D). EM = egg mass. CS = clutch size, number of eggs per clutch. CM/ACM = clutch mass (CM; CM = EM x CS) respectively annual clutch mass (ACM). ACM equals CM because as a first approximation we assumed one clutch per year for all dinosaurs studied. Values in brackets are minimum and maximum values, where no maxima are given only one value was available. Note: the mean BMs for both titanosaur taxa were established from body masses of sauropods larger than 5000 kg (because sauropod species with a BM of at least 5000 kg were assigned to both *Megaloolithus* oospecies) and for which the body mass estimation method was given in the source. The exact body mass range is 6853 kg to 72936 kg, however we assumed, as a somewhat more conservative measure, minimum and maximum body masses of 5000 kg and of 75000 kg.
(DOCX)

Table S2 **Data used in the allometric analyses of extant species.** G = Galliformes, A = Anseriformes, S = Struthioniformes, C = Crocodylia, T = Testudinidae, BM = body mass, EM = egg mass, CM = clutch mass, ACM = annual clutch mass.
(DOCX)

Table S3 **Results of the OLS regression analyses carried out for body mass against several reproductive traits for tortoises, crocodiles and birds.** Allometric functions follow *c*×BM^b^ (c+b×BM, log-log-plot), where BM is the body mass in kilograms, *c* is the intercept and *b* the slope of the line in a log-log-plot. N = sample size. 95% CI = 95% confidence interval of *c* or *b*. EM = egg mass. CM = clutch mass. ACM = annual clutch mass.
(DOCX)

Table S4 **Summary of results obtained in ANCOVAs.** With these analyses we tested for differences in regression slopes ((a); for EM, CM, ACM) and intercepts ((b); CM, ACM) obtained for birds, crocodiles and tortoises under ordinary least squares regression analysis.
EM = egg mass, CM = clutch mass, ACM = annual clutch mass, group = categorical variable, coding whether the species is a bird, crocodile or tortoises, log = logarithm to the base 10. n.a. = not applicable. For sample sizes refer to Table S3.
(DOCX)

Table S5 **Summary of results obtained in ANCOVAs.** With these analyses we tested for differences in regression slopes ((a); for EM, CM, ACM) and intercepts ((b); CM, ACM) obtained for crocodiles and tortoises under ordinary least squares regression analysis.
EM = egg mass, CM = clutch mass, ACM = annual clutch mass, group = categorical variable, coding whether the species is a crocodile or tortoises, log = logarithm to the base 10. n.a. = not applicable. For sample sizes refer to Table S3.
(DOCX)

Table S6 **Allometric models used to estimate reproductive traits of non-avian dinosaurs.** EM = egg mass, CM = clutch mass, ACM = annual clutch mass; c = intercept and b = slope of the respective allometry.
(DOCX)
