# Supplementary material for: New Insights into Non-Avian Dinosaur Reproduction and Their Evolutionary and Ecological Implications: Linking Fossil Evidence to Allometries of Extant Close Relatives
Source: PLoS One. 2013 Aug 21;8(8):e72862. doi: 10.1371/journal.pone.0072862 (PMC3749170; doi:10.1371/journal.pone.0072862)
Supplement: Table S1 — Average body mass and reproductive characteristics of dinosaurs as documented in the fossil record. The Megaloolithus patagonicus oospecies is assigned to a titanosaurian sauropod based on embryonic remains in the eggs, and the Megaloolithussiruguei /mammilare egg type (with a highly porous shell) is commonly assigned to titanosaurian sauropod dinosaurs, because titanosaur bones had been found in the same horizon or formation as the eggs. It should be noted that Megaloolithussiruguei is considered as a junior synonym of Megaloolithusmammillare . Megaloolithus eggs have also been assigned to titanosaurs because of the find of a hatchling in a nest of Megaloolitgus eggs from India. Although taxonomic identification of the eggs and their producers is problematic in sauropods, species with a mass of at least 5000 kg were assigned to both Megaloolithus oospecies. BM = body mass. ES = egg size, expressed in length (L) and diameter (D). EM = egg mass. CS = clutch size, number of eggs per clutch. CM/ACM = clutch mass (CM; CM = EM x CS) respectively annual clutch mass (ACM). ACM equals CM because as a first approximation we assumed one clutch per year for all dinosaurs studied. Values in brackets are minimum and maximum values, where no maxima are given only one value was available. Note: the mean BMs for both titanosaur taxa were established from body masses of sauropods larger than 5000 kg (because sauropod species with a BM of at least 5000 kg were assigned to both Megaloolithus oospecies) and for which the body mass estimation method was given in the source. The exact body mass range is 6853 kg to 72936 kg, however we assumed, as a somewhat more conservative measure, minimum and maximum body masses of 5000 kg and of 75000 kg. (DOCX) [file pone.0072862.s001.docx]

**Table S1.** **Average body mass and reproductive characteristics of dinosaurs as documented in the fossil record.** The *Megaloolithus patagonicus* oospecies is assigned to a titanosaurian sauropod based on embryonic remains in the eggs [[1-4](#_ENREF_1)], and the *Megaloolithus siruguei/mammilare* egg type (with a highly porous shell) is commonly assigned to titanosaurian sauropod dinosaurs, because titanosaur bones had been found in the same horizon or formation as the eggs. It should be noted that *Megaloolithus siruguei* is considered as a junior synonym of *Megaloolithus mammillare* [[5-7](#_ENREF_5)]. Megaloolithus eggs have also been assigned to titanosaurs because of the find of a hatchling in a nest of Megaloolitgus eggs from India[[8](#_ENREF_8)]. Although taxonomic identification of the eggs and their producers is problematic in sauropods, species with a mass of at least 5000 kg were assigned to both *Megaloolithus* oospecies [[7](#_ENREF_7),[9](#_ENREF_9)]. BM = body mass. ES = egg size, expressed in length (L) and diameter (D). EM = egg mass. CS = clutch size, number of eggs per clutch. CM/ACM = clutch mass (CM; CM = EM x CS) respectively annual clutch mass (ACM). ACM equals CM because as a first approximation we assumed one clutch per year for all dinosaurs studied. Values in brackets are minimum and maximum values, where no maxima are given only one value was available.

| **Taxon** | **BM (kg)** | **ES (cm)** | | **EM (kg)** | **CS (#)** | **CM/ACM (kg)** | **References** |
| --- | --- | --- | --- | --- | --- | --- | --- |
|  |  | **L** | **D** |  |  |  |  |
| *Troodoon formosus* | 44 [34, 51] | 13.5 | 6.5 | 0.329 | 23 [22, 24] | 7.567 [7.238, 7.896] | [[9-13](#_ENREF_9)] |
| *Oviraptor philoceratops* | 37 [33, 40] | 15.0 | 5.5 | 0.262 | 24 [20, 30] | 6.288 [5.240, 7.860] | [[9](#_ENREF_9),[10](#_ENREF_10),[13-15](#_ENREF_13)] |
| *Citipati osmolskae* | 79 | 18.3 | 6.7 | 0.473 | 22 [15, 30] | 10.406 [7.095, 14.190] | [[9](#_ENREF_9),[14](#_ENREF_14),[16](#_ENREF_16)] |
| *Lourinhanosaurus antunesi* | 176 | 12.9 | 9.0 | 0.602 | 63 [25, 100] | 37.926 [15.050, 60.200] | [[17](#_ENREF_17),[18](#_ENREF_18)] |
| *lambeosaurine dinosaur* | 3344 [2390, 5057] | 20.0 | 20.0 | 4.737 | 22 | 104.214 | [[19-23](#_ENREF_19)] |
| *Maiasaura peeblesorum* | 2556 [1500, 4079] | 12.0 | 12.0 | 1.023 | 16 | 16.368 | [[21](#_ENREF_21),[24](#_ENREF_24),[25](#_ENREF_25)] |
| *Massospondylus carinatus* | 175 [107, 280] | 6.0 | 6.0 | 0.128 | 34 | 4.352 | [[9](#_ENREF_9),[22](#_ENREF_22),[24](#_ENREF_24),[26](#_ENREF_26),[27](#_ENREF_27)] |
| *titanosaur (Megaloolithus patagonicus)* | 22399 [5000, 75000] | 15.0 | 14.0 | 1.741 | 28 [15, 40] | 48.748 [26.115, 69.640] | [[28](#_ENREF_28),[29](#_ENREF_29)] |
| *titanosaur (Megaloolithus mammilare)* | 22399 [5000, 75000] | 22.0 | 20.0 | 5.211 | 19 [9, 28] | 99.009 [46.899, 145.908] | [[28-30](#_ENREF_28)] |

Note: the mean BMs for both *titanosaur* taxa were established from body masses of sauropods larger than 5000 kg (because sauropod species with a BM of at least 5000 kg were assigned to both *Megaloolithus* oospecies [[7](#_ENREF_7),[9](#_ENREF_9)]) and for which the body mass estimation method was given in the source. The exact body mass range is 6853 kg to 72936 kg, however we assumed, as a somewhat more conservative measure, minimum and maximum body masses of 5000 kg and of 75000 kg.

**References**

1. Chiappe LM, Coria RA, Dingus L, Jackson F, Chinsamy A, et al. (1998) Sauropod dinosaur embryos from the late Cretaceous of Patagonia. Nature 396: 258-261.

2. Chiappe LM, Salgado L, Coria RA (2001) Embryonic skulls of titanosaur sauropod dinosaurs. Science 293: 2444-2446.

3. García RA, Cerda IA (2010) Dentition and histology in titanosaurian dinosaur embryos from Upper Cretaceous of Patagonia, Argentina. Palaeontology 53: 335-346.

4. Grellet-Tinner G, Chiappe LM, Coria R (2004) Eggs of titanosaurid sauropods from the Upper Cretaceous of Auca Mahuevo (Argentina). Can J Earth Sci 41: 949-960.

5. Peitz C (1999) Parataxonomic implications of some megaloolithid dinosaur eggs from Catalunya, Spain. In: Bravo AM, Reyes T, editors. First international symposium on dinosaur eggs and babies, extended abstracts. Isona i Conda Dellà, Spain. pp. 49–50.

6. Peitz C (2000) Megaloolithid dinosaur eggs from Maastrichtian of Catalunya (NE-Spain): parataxonomic implications and stratigraphic utility. In: Bravo AM, Reyes T, editors. International symposium on dinosaur eggs and babies, extended abstracts. Isona i Conda Dellà, Spain. pp. 155-159.

7. Sander PM, Peitz C, Jackson FD, Chiappe LM (2008) Upper Cretaceous titanosaur nesting sites and their implications for sauropod dinosaur reproductive biology. Palaeontogr Abt A 284: 69-107.

8. Wilson JA, Mohabey DM, Peters SE, Head JJ (2010) Predation upon hatchling dinosaurs by a new snake from the Late Cretaceous of India. PLoS Biol 8: e1000322.

9. Varricchio DJ, Moore JR, Erickson GM, Norell MA, Jackson FD, et al. (2008) Avian paternal care had dinosaur origin. Science 322: 1826-1828.

10. Peczkis J (1994) Implications of body-mass estimates for dinosaurs. J Vert Paleontol 14: 520-533.

11. Russell DA (1969) A new specimen of *Stenonychosaurus* from the Oldman Formation (Cretaceous) of Alberta. Can J Earth Sci 6: 595-612.

12. Russell DA, Séguin R (1982) Reconstruction of the small Cretaceous theropod *Stenonychosaurus inequalis* and a hypothetical dinosauroid. Syllogeus 37: 1-43.

13. Paul GS (1988) Predatory dinosaurs of the world. New York: Simon and Schuster. 464 p.

14. Norell MA, Clark JM, Chiappe LM, Dashzeveg D (1995) A nesting dinosaur. Nature 378: 774-776.

15. Mikhailov K, Sabath K, Kurzanov S (1994) Eggs and nests from the Cretaceous of Mongolia. In: Carpenter K, Hirsch KF, Horner JR, editors. Dinosaur eggs and babies. Cambridge: Cambridge University Press.

16. Clark JM, Norell MA, Chiappe LM (1999) An oviraptorid skeleton from the Late Cretaceous of Ukhaa Tolgod, Mongolia, preserved in an avianlike brooding position over an oviraptorid nest. Am Mus Novit 3265: 1-36.

17. Mateus I, Mateus H, Antunes MT, Mateus O, Taquet P, et al. (1998) Upper Jurassic theropod dinosaur embryos from Lourinhã (Portugal). Mem Acad Ci Lisboa 37: 101-109.

18. Mateus IH, Antunes MT, Mateus O, Tacquet P, Ribeiro V, et al. (1997) Couvée, oeufs et embryons d'un dinosaure théropode du Jurassique supérieur de Lourinhã (Portugal). C R Acad Sci Paris 325: 71-78.

19. Amadon D (1947) An estimated weight of the largest known bird. Condor 49: 159-164.

20. Paleobiology, Database (2011) Lambeosaurus. The data were downloaded from the Paleobiology Database on 5 Juli 2011.

21. Horner JR (1999) Egg clutches and embryos of two hadrosaurian dinosaurs. J Vert Paleontol 19: 607-611.

22. Seebacher F (2001) A new method to calculate allometric length-mass relationships of dinosaurs. J Vert Paleontol 21: 51-60.

23. Evans DC, Ridgely R, Witmer LM (2009) Endocranial anatomy of lambeosaurine Hadrosaurids (Dinosauria:Ornithischia): a sensorineural perspective on cranial crest function. Anat Rec 292: 1315-1337.

24. Erickson GM, Rogers KC, Yerby SA (2001) Dinosaurian growth patterns and rapid avian growth rates. Letters to Nature 412: 429-433.

25. Seebacher F (2003) Dinosaur body temperatures: the occurrence of endothermy and ectothermy. Paleobiology 29: 105-122.

26. Reisz RR, Evans DC, Roberts EM, Sues H-D, Yates AM (2012) Oldest known dinosaurian nesting site and reproductive biology of the Early Jurassic sauropodomorph *Massospondylus*. Proc Natl Acad Sci USA 109: 2428-2433.

27. Reisz RR, Scott D, Sues HD, Evans DC, Raath MA (2005) Embryos of an Early Jurassic prosauropod dinosaur and their evolutionary significance. Science 309: 761-764.

28. Jackson FD, Varricchio DJ, Jackson RA, Vila B, Chiappe LM (2008) Comparison of water vapor conductance in a titanosaur egg from the Upper Cretaceous of Argentina and a *Megaloolithus siruguei* egg from Spain. Paleobiology 34: 229-246.

29. Sander PM, Christian A, Clauss M, Fechner R, Gee CT, et al. (2011) Biology of the sauropod dinosaurs: the evolution of gigantism. Biol Rev 86: 117-155.

30. Vila B, Jackson FD, Fortuny J, Sellés AG, Galobart À (2010) 3-D modelling of megaloolithid clutches: insights about nest construction and dinosaur behaviour. PLoS ONE 5: e10362.
