# Supplementary material for: New Insights into Non-Avian Dinosaur Reproduction and Their Evolutionary and Ecological Implications: Linking Fossil Evidence to Allometries of Extant Close Relatives
Source: PLoS One. 2013 Aug 21;8(8):e72862. doi: 10.1371/journal.pone.0072862 (PMC3749170; doi:10.1371/journal.pone.0072862)
Supplement: Table S2 — Data used in the allometric analyses of extant species. G = Galliformes, A = Anseriformes, S = Struthioniformes, C = Crocodylia, T = Testudinidae, BM = body mass, EM = egg mass, CM = clutch mass, ACM = annual clutch mass. (DOCX) [file pone.0072862.s002.docx]

**Table S2.** Data used in the allometric analyses of extant species. G = Galliformes, A = Anseriformes, S = Struthioniformes, C = Crocodylia, T = Testudinidae, BM = body mass, EM = egg mass, CM = clutch mass, ACM = annual clutch mass.

| **Species** | **Group** | **BM** | **EM** | **CM** | **ACM** | **References** |
| --- | --- | --- | --- | --- | --- | --- |
| *Alectoris barbara* | G | 0.3760 | 0.0196 | 0.2352 | 0.3528 | [[1](#_ENREF_1),[2](#_ENREF_2)] |
| *Alectoris chukar* | G | 0.5010 | 0.0225 | 0.3488 | 0.5231 | [[2](#_ENREF_2),[3](#_ENREF_3)] |
| *Alectoris graeca* | G | 0.5750 | 0.0197 | 0.2266 | 0.3398 | [[1](#_ENREF_1),[2](#_ENREF_2)] |
| *Alectoris rufa* | G | 0.4390 | 0.0201 | 0.2553 | 0.3829 | [[2](#_ENREF_2),[3](#_ENREF_3)] |
| *Bambusicola thoracica* | G | 0.2710 | 0.0119 | 0.0595 | 0.0595 | [[2](#_ENREF_2),[3](#_ENREF_3)] |
| *Bonasa bonasia* | G | 0.3700 | 0.0190 | 0.1782 | 0.1782 | [[2](#_ENREF_2),[3](#_ENREF_3)] |
| *Bonasa umbellus* | G | 0.4720 | 0.0180 | 0.2070 | 0.2070 | [[2](#_ENREF_2),[3](#_ENREF_3)] |
| *Catreus wallichii* | G | 1.3050 | 0.0716 | 0.7518 | 0.7518 | [[2](#_ENREF_2),[3](#_ENREF_3)] |
| *Centrocercus urophasianus* | G | 1.7000 | 0.0461 | 0.3347 | 0.3347 | [[2](#_ENREF_2),[3](#_ENREF_3)] |
| *Chrysolophus amherstiae* | G | 0.7140 | 0.0311 | 0.2799 | 0.2799 | [[2](#_ENREF_2),[3](#_ENREF_3)] |
| *Chrysolophus pictus* | G | 0.6075 | 0.0267 | 0.2403 | 0.2403 | [[2](#_ENREF_2),[3](#_ENREF_3)] |
| *Coturnix chinensis* | G | 0.0356 | 0.0048 | 0.0360 | 0.0360 | [[2](#_ENREF_2),[3](#_ENREF_3)] |
| *Coturnix coturnix* | G | 0.1030 | 0.0082 | 0.0845 | 0.0845 | [[2](#_ENREF_2),[3](#_ENREF_3)] |
| *Coturnix delegorguei* | G | 0.0785 | 0.0075 | 0.0360 | 0.0360 | [[2](#_ENREF_2),[3](#_ENREF_3)] |
| *Coturnix pectoralis* | G | 0.1060 | 0.0086 | 0.0903 | 0.0903 | [[2](#_ENREF_2),[3](#_ENREF_3)] |
| *Coturnix ypsilophora* | G | 0.1030 | 0.0112 | 0.0885 | 0.1327 | [[2](#_ENREF_2),[3](#_ENREF_3)] |
| *Crossoptilon auritum* | G | 1.6000 | 0.0630 | 0.4095 | 0.4095 | [[2](#_ENREF_2),[3](#_ENREF_3)] |
| *Crossoptilon crossoptilon* | G | 1.6750 | 0.0532 | 0.3940 | 0.7880 | [[2](#_ENREF_2),[3](#_ENREF_3)] |
| *Dendragapus canadensis* | G | 0.4240 | 0.0228 | 0.1368 | 0.1368 | [[2](#_ENREF_2),[3](#_ENREF_3)] |
| *Dendragapus falcipennis* | G | 0.6950 | 0.0260 | 0.2990 | 0.2990 | [[2](#_ENREF_2),[3](#_ENREF_3)] |
| *Dendragapus obscurus* | G | 0.8390 | 0.0325 | 0.2763 | 0.2763 | [[2](#_ENREF_2),[3](#_ENREF_3)] |
| *Francolinus adspersus* | G | 0.3940 | 0.0268 | 0.1796 | 0.1796 | [[2](#_ENREF_2),[3](#_ENREF_3)] |
| *Francolinus africanus* | G | 0.3975 | 0.0178 | 0.1282 | 0.1922 | [[2](#_ENREF_2),[3](#_ENREF_3)] |
| *Francolinus albogularis* | G | 0.2735 | 0.0140 | 0.0770 | 0.0770 | [[2](#_ENREF_2),[3](#_ENREF_3)] |
| *Francolinus bicalcaratus* | G | 0.3810 | 0.0265 | 0.1458 | 0.1458 | [[2](#_ENREF_2),[3](#_ENREF_3)] |
| *Francolinus capensis* | G | 0.5470 | 0.0330 | 0.2442 | 0.2442 | [[2](#_ENREF_2),[3](#_ENREF_3)] |
| *Francolinus castaneicollis* | G | 0.6000 | 0.0375 | 0.2063 | 0.2063 | [[2](#_ENREF_2),[3](#_ENREF_3)] |
| *Francolinus francolinus* | G | 0.4250 | 0.0235 | 0.1293 | 0.1939 | [[2](#_ENREF_2),[3](#_ENREF_3)] |
| *Francolinus icterorhynchus* | G | 0.4410 | 0.0199 | 0.1393 | 0.1393 | [[2](#_ENREF_2),[3](#_ENREF_3)] |
| *Francolinus levaillantii* | G | 0.4010 | 0.0210 | 0.1050 | 0.1050 | [[2](#_ENREF_2),[3](#_ENREF_3)] |
| *Francolinus levaillantoides* | G | 0.4145 | 0.0188 | 0.1125 | 0.1125 | [[2](#_ENREF_2),[3](#_ENREF_3)] |
| *Francolinus natalensis* | G | 0.3850 | 0.0270 | 0.1755 | 0.1755 | [[2](#_ENREF_2),[3](#_ENREF_3)] |
| *Francolinus pondicerianus* | G | 0.2280 | 0.0127 | 0.0953 | 0.1429 | [[1](#_ENREF_1),[2](#_ENREF_2)] |
| *Francolinus shelleyi* | G | 0.4260 | 0.0173 | 0.0830 | 0.1246 | [[1](#_ENREF_1),[2](#_ENREF_2)] |
| *Francolinus swainsonii* | G | 0.5100 | 0.0291 | 0.1601 | 0.2401 | [[2](#_ENREF_2),[3](#_ENREF_3)] |
| *Gallus gallus* | G | 0.7675 | 0.0296 | 0.1776 | 0.1776 | [[2](#_ENREF_2),[3](#_ENREF_3)] |
| *Gallus lafayetii* | G | 0.5675 | 0.0304 | 0.0912 | 0.0912 | [[2](#_ENREF_2),[3](#_ENREF_3)] |
| *Gallus sonneratii* | G | 0.7475 | 0.0334 | 0.1503 | 0.1503 | [[2](#_ENREF_2),[3](#_ENREF_3)] |
| *Gallus varius* | G | 0.7675 | 0.0292 | 0.1022 | 0.1022 | [[2](#_ENREF_2),[3](#_ENREF_3)] |
| *Ithaginis cruentus* | G | 0.5150 | 0.0288 | 0.2448 | 0.2448 | [[2](#_ENREF_2),[3](#_ENREF_3)] |
| *Lagopus lagopus* | G | 0.5550 | 0.0215 | 0.2043 | 0.2043 | [[2](#_ENREF_2),[3](#_ENREF_3)] |
| *Lagopus leucurus* | G | 0.3510 | 0.0200 | 0.1180 | 0.1180 | [[2](#_ENREF_2),[3](#_ENREF_3)] |
| *Lagopus mutus* | G | 0.4140 | 0.0200 | 0.1700 | 0.1700 | [[2](#_ENREF_2),[3](#_ENREF_3)] |
| *Lophophorus impejanus* | G | 1.9750 | 0.0707 | 0.4242 | 0.4242 | [[2](#_ENREF_2),[3](#_ENREF_3)] |
| *Lophura leucomelanos* | G | 0.7940 | 0.0374 | 0.2805 | 0.2805 | [[2](#_ENREF_2),[3](#_ENREF_3)] |
| *Lophura nycthemera* | G | 1.1300 | 0.0428 | 0.2140 | 0.2140 | [[2](#_ENREF_2),[3](#_ENREF_3)] |
| *Meleagris gallopavo* | G | 4.2220 | 0.0788 | 0.9850 | 0.9850 | [[2](#_ENREF_2),[3](#_ENREF_3)] |
| *Pavo cristatus* | G | 3.3750 | 0.1035 | 0.6210 | 0.6210 | [[2](#_ENREF_2),[3](#_ENREF_3)] |
| *Perdix dauurica* | G | 0.2700 | 0.0127 | 0.2286 | 0.2286 | [[2](#_ENREF_2),[3](#_ENREF_3)] |
| *Perdix perdix* | G | 0.3860 | 0.0145 | 0.2117 | 0.2117 | [[2](#_ENREF_2),[3](#_ENREF_3)] |
| *Phasianus colchicus* | G | 0.9890 | 0.0315 | 0.3465 | 0.3465 | [[2](#_ENREF_2),[3](#_ENREF_3)] |
| *Pucrasia macrolopha* | G | 0.9320 | 0.0400 | 0.2400 | 0.2400 | [[2](#_ENREF_2),[3](#_ENREF_3)] |
| *Syrmaticus reevesii* | G | 0.9490 | 0.0348 | 0.3306 | 0.3306 | [[2](#_ENREF_2),[3](#_ENREF_3)] |
| *Tetrao mlokosiewiczi* | G | 0.7660 | 0.0325 | 0.1983 | 0.1983 | [[2](#_ENREF_2),[3](#_ENREF_3)] |
| *Tetrao parvirostris* | G | 1.9500 | 0.0540 | 0.3510 | 0.3510 | [[2](#_ENREF_2),[3](#_ENREF_3)] |
| *Tetrao tetrix* | G | 0.9880 | 0.0355 | 0.2808 | 0.2808 | [[2](#_ENREF_2),[3](#_ENREF_3)] |
| *Tetrao urogallus* | G | 1.9850 | 0.0530 | 0.3286 | 0.3286 | [[2](#_ENREF_2),[3](#_ENREF_3)] |
| *Tympanuchus cupido* | G | 0.7950 | 0.0245 | 0.2940 | 0.2940 | [[2](#_ENREF_2),[3](#_ENREF_3)] |
| *Tympanuchus pallidicinctus* | G | 0.6280 | 0.0240 | 0.2568 | 0.2568 | [[2](#_ENREF_2),[3](#_ENREF_3)] |
| *Tympanuchus phasianellus* | G | 0.8170 | 0.0240 | 0.2616 | 0.2616 | [[2](#_ENREF_2),[3](#_ENREF_3)] |
| *Aix galericulata* | A | 0.5120 | 0.0387 | 0.3599 | 0.3599 | [[4](#_ENREF_4),[5](#_ENREF_5)] |
| *Aix sponsa* | A | 0.6350 | 0.0426 | 0.4729 | 0.4729 | [[4](#_ENREF_4),[5](#_ENREF_5)] |
| *Alopochen aegyptiacus* | A | 1.6500 | 0.0957 | 0.8135 | 0.8135 | [[4](#_ENREF_4),[5](#_ENREF_5)] |
| *Amazonetta brasiliensis* | A | 0.3700 | 0.0333 | 0.2331 | 0.2331 | [[4](#_ENREF_4),[5](#_ENREF_5)] |
| *Anas acuta* | A | 0.9860 | 0.0403 | 0.2781 | 0.2781 | [[4](#_ENREF_4),[5](#_ENREF_5)] |
| *Anas americana* | A | 0.7190 | 0.0441 | 0.3749 | 0.3749 | [[4](#_ENREF_4),[5](#_ENREF_5)] |
| *Anas aucklandica* | A | 0.4100 | 0.0705 | 0.2820 | 0.2820 | [[4](#_ENREF_4),[5](#_ENREF_5)] |
| *Anas bahamensis* | A | 0.5690 | 0.0405 | 0.3402 | 0.3402 | [[4](#_ENREF_4),[5](#_ENREF_5)] |
| *Anas bernieri* | A | 0.3440 | 0.0286 | 0.1802 | 0.1802 | [[4](#_ENREF_4),[5](#_ENREF_5)] |
| *Anas capensis* | A | 0.3800 | 0.0359 | 0.3016 | 0.3016 | [[4](#_ENREF_4),[5](#_ENREF_5)] |
| *Anas carolinensis* | A | 0.3090 | 0.0252 | 0.2167 | 0.2167 | [[4](#_ENREF_4),[5](#_ENREF_5)] |
| *Anas castanea* | A | 0.5900 | 0.0440 | 0.4268 | 0.4268 | [[4](#_ENREF_4),[5](#_ENREF_5)] |
| *Anas chlorotis* | A | 0.4790 | 0.0608 | 0.3344 | 0.3344 | [[4](#_ENREF_4),[5](#_ENREF_5)] |
| *Anas clypeata* | A | 0.5900 | 0.0391 | 0.3988 | 0.3988 | [[4](#_ENREF_4),[5](#_ENREF_5)] |
| *Anas crecca* | A | 0.3180 | 0.0290 | 0.2755 | 0.2755 | [[4](#_ENREF_4),[5](#_ENREF_5)] |
| *Anas cyanoptera* | A | 0.3630 | 0.0308 | 0.2988 | 0.2988 | [[4](#_ENREF_4),[5](#_ENREF_5)] |
| *Anas discors* | A | 0.3630 | 0.0281 | 0.2922 | 0.2922 | [[4](#_ENREF_4),[5](#_ENREF_5)] |
| *Anas eatoni* | A | 0.4410 | 0.0396 | 0.1980 | 0.1980 | [[4](#_ENREF_4),[5](#_ENREF_5)] |
| *Anas erythrorhyncha* | A | 0.5660 | 0.0401 | 0.3609 | 0.3609 | [[4](#_ENREF_4),[5](#_ENREF_5)] |
| *Anas falcata* | A | 0.5850 | 0.0497 | 0.3976 | 0.3976 | [[4](#_ENREF_4),[5](#_ENREF_5)] |
| *Anas flavirostris* | A | 0.3940 | 0.0343 | 0.2230 | 0.2230 | [[4](#_ENREF_4),[5](#_ENREF_5)] |
| *Anas formosa* | A | 0.4310 | 0.0309 | 0.2256 | 0.2256 | [[4](#_ENREF_4),[5](#_ENREF_5)] |
| *Anas fulvigula* | A | 0.9680 | 0.0500 | 0.5000 | 0.5000 | [[4](#_ENREF_4),[5](#_ENREF_5)] |
| *Anas georgica* | A | 0.5350 | 0.0370 | 0.1554 | 0.1554 | [[4](#_ENREF_4),[5](#_ENREF_5)] |
| *Anas gibberifrons* | A | 0.4690 | 0.0360 | 0.2844 | 0.2844 | [[4](#_ENREF_4),[5](#_ENREF_5)] |
| *Anas hottentata* | A | 0.2400 | 0.0266 | 0.1889 | 0.1889 | [[4](#_ENREF_4),[5](#_ENREF_5)] |
| *Anas laysanensis* | A | 0.4270 | 0.0441 | 0.1499 | 0.1499 | [[4](#_ENREF_4),[5](#_ENREF_5)] |
| *Anas luzonica* | A | 0.7790 | 0.0504 | 0.5040 | 0.5040 | [[4](#_ENREF_4),[5](#_ENREF_5)] |
| *Anas melleri* | A | 0.9110 | 0.0501 | 0.4083 | 0.4083 | [[4](#_ENREF_4),[5](#_ENREF_5)] |
| *Anas penelope* | A | 0.7240 | 0.0464 | 0.4176 | 0.4176 | [[4](#_ENREF_4),[5](#_ENREF_5)] |
| *Anas platalea* | A | 0.5230 | 0.0413 | 0.2685 | 0.2685 | [[4](#_ENREF_4),[5](#_ENREF_5)] |
| *Anas platyrhynchos* | A | 1.1230 | 0.0499 | 0.4840 | 0.4840 | [[4](#_ENREF_4),[5](#_ENREF_5)] |
| *Anas poecilorhyncha* | A | 1.0250 | 0.0556 | 0.4726 | 0.4726 | [[4](#_ENREF_4),[5](#_ENREF_5)] |
| *Anas querquedula* | A | 0.3100 | 0.0280 | 0.2380 | 0.2380 | [[4](#_ENREF_4),[5](#_ENREF_5)] |
| *Anas rubripes* | A | 1.1000 | 0.0615 | 0.5843 | 0.5843 | [[4](#_ENREF_4),[5](#_ENREF_5)] |
| *Anas sibilatrix* | A | 0.8280 | 0.0572 | 0.3718 | 0.3718 | [[4](#_ENREF_4),[5](#_ENREF_5)] |
| *Anas smithii* | A | 0.5970 | 0.0447 | 0.4202 | 0.4202 | [[4](#_ENREF_4),[5](#_ENREF_5)] |
| *Anas sparsa* | A | 0.9140 | 0.0677 | 0.3994 | 0.3994 | [[4](#_ENREF_4),[5](#_ENREF_5)] |
| *Anas strepera* | A | 0.8490 | 0.0459 | 0.4361 | 0.4361 | [[4](#_ENREF_4),[5](#_ENREF_5)] |
| *Anas superciliosa* | A | 0.9810 | 0.0541 | 0.4923 | 0.4923 | [[4](#_ENREF_4),[5](#_ENREF_5)] |
| *Anas undulata* | A | 0.8170 | 0.0524 | 0.4087 | 0.4087 | [[4](#_ENREF_4),[5](#_ENREF_5)] |
| *Anas versicolor* | A | 0.3730 | 0.0306 | 0.2601 | 0.2601 | [[4](#_ENREF_4),[5](#_ENREF_5)] |
| *Anas wyvilliana* | A | 0.5850 | 0.0321 | 0.2664 | 0.2664 | [[4](#_ENREF_4),[5](#_ENREF_5)] |
| *Anser albifrons* | A | 2.4560 | 0.1280 | 0.6272 | 0.6272 | [[4](#_ENREF_4),[5](#_ENREF_5)] |
| *Anser anser* | A | 3.1080 | 0.1650 | 0.9735 | 0.9735 | [[4](#_ENREF_4),[5](#_ENREF_5)] |
| *Anser brachyrhynchus* | A | 2.5200 | 0.1225 | 0.5268 | 0.5268 | [[4](#_ENREF_4),[5](#_ENREF_5)] |
| *Anser caerulescens* | A | 2.5170 | 0.1220 | 0.4880 | 0.4880 | [[4](#_ENREF_4),[5](#_ENREF_5)] |
| *Anser canagicus* | A | 2.7660 | 0.1204 | 0.5779 | 0.5779 | [[4](#_ENREF_4),[5](#_ENREF_5)] |
| *Anser cygnoides* | A | 3.1500 | 0.1427 | 0.7849 | 0.7849 | [[4](#_ENREF_4),[5](#_ENREF_5)] |
| *Anser erythropus* | A | 1.7250 | 0.1030 | 0.5150 | 0.5150 | [[4](#_ENREF_4),[5](#_ENREF_5)] |
| *Anser fabalis* | A | 2.8430 | 0.1462 | 0.7310 | 0.7310 | [[4](#_ENREF_4),[5](#_ENREF_5)] |
| *Anser indicus* | A | 2.2300 | 0.1422 | 0.7110 | 0.7110 | [[4](#_ENREF_4),[5](#_ENREF_5)] |
| *Anser rossii* | A | 1.5000 | 0.0915 | 0.3477 | 0.3477 | [[4](#_ENREF_4),[5](#_ENREF_5)] |
| *Anseranas semipalmata* | A | 2.0710 | 0.1122 | 0.9649 | 0.9649 | [[4](#_ENREF_4),[5](#_ENREF_5)] |
| *Aythya affinis* | A | 0.7900 | 0.0482 | 0.4916 | 0.4916 | [[4](#_ENREF_4),[5](#_ENREF_5)] |
| *Aythya americana* | A | 0.9900 | 0.0629 | 0.5913 | 0.5913 | [[4](#_ENREF_4),[5](#_ENREF_5)] |
| *Aythya australis* | A | 0.8380 | 0.0558 | 0.5580 | 0.5580 | [[4](#_ENREF_4),[5](#_ENREF_5)] |
| *Aythya baeri* | A | 0.6800 | 0.0409 | 0.4090 | 0.4090 | [[4](#_ENREF_4),[5](#_ENREF_5)] |
| *Aythya collaris* | A | 0.6800 | 0.0499 | 0.4741 | 0.4741 | [[4](#_ENREF_4),[5](#_ENREF_5)] |
| *Aythya ferina* | A | 0.8070 | 0.0680 | 0.5644 | 0.5644 | [[4](#_ENREF_4),[5](#_ENREF_5)] |
| *Aythya fuligula* | A | 0.6800 | 0.0555 | 0.5328 | 0.5328 | [[4](#_ENREF_4),[5](#_ENREF_5)] |
| *Aythya marila* | A | 0.9570 | 0.0661 | 0.6412 | 0.6412 | [[4](#_ENREF_4),[5](#_ENREF_5)] |
| *Aythya novaeseelandiae* | A | 0.6870 | 0.0597 | 0.4179 | 0.4179 | [[4](#_ENREF_4),[5](#_ENREF_5)] |
| *Aythya nyroca* | A | 0.5200 | 0.0425 | 0.3825 | 0.3825 | [[4](#_ENREF_4),[5](#_ENREF_5)] |
| *Aythya valisineria* | A | 1.1900 | 0.0705 | 0.5781 | 0.5781 | [[4](#_ENREF_4),[5](#_ENREF_5)] |
| *Biziura lobata* | A | 1.5510 | 0.1279 | 0.3581 | 0.3581 | [[4](#_ENREF_4),[5](#_ENREF_5)] |
| *Branta bernicla* | A | 1.2300 | 0.0910 | 0.3640 | 0.3640 | [[4](#_ENREF_4),[5](#_ENREF_5)] |
| *Branta canadiensis* | A | 3.3140 | 0.1690 | 0.9464 | 0.9464 | [[4](#_ENREF_4),[5](#_ENREF_5)] |
| *Branta hrota* | A | 1.0900 | 0.0840 | 0.3276 | 0.3276 | [[4](#_ENREF_4),[5](#_ENREF_5)] |
| *Branta leucopsis* | A | 1.5860 | 0.1040 | 0.4680 | 0.4680 | [[4](#_ENREF_4),[5](#_ENREF_5)] |
| *Branta ruficollis* | A | 1.0940 | 0.0782 | 0.3519 | 0.3519 | [[4](#_ENREF_4),[5](#_ENREF_5)] |
| *Branta sandvicensis* | A | 1.9300 | 0.1440 | 0.6048 | 0.6048 | [[4](#_ENREF_4),[5](#_ENREF_5)] |
| *Bucephala albeola* | A | 0.3340 | 0.0367 | 0.3230 | 0.3230 | [[4](#_ENREF_4),[5](#_ENREF_5)] |
| *Bucephala clangula* | A | 0.8000 | 0.0641 | 0.5577 | 0.5577 | [[4](#_ENREF_4),[5](#_ENREF_5)] |
| *Bucephala islandica* | A | 0.7300 | 0.0677 | 0.5348 | 0.5348 | [[4](#_ENREF_4),[5](#_ENREF_5)] |
| *Cairina moschata* | A | 2.0220 | 0.0787 | 0.6926 | 0.6926 | [[4](#_ENREF_4),[5](#_ENREF_5)] |
| *Cairina scutulata* | A | 2.6000 | 0.0890 | 0.8900 | 0.8900 | [[4](#_ENREF_4),[5](#_ENREF_5)] |
| *Callonetta leucophrys* | A | 0.3210 | 0.0324 | 0.2916 | 0.2916 | [[4](#_ENREF_4),[5](#_ENREF_5)] |
| *Cereopsis novaehollandiae* | A | 3.7700 | 0.1269 | 0.5203 | 0.5203 | [[4](#_ENREF_4),[5](#_ENREF_5)] |
| *Chenonetta jubata* | A | 0.8000 | 0.0558 | 0.5580 | 0.5580 | [[4](#_ENREF_4),[5](#_ENREF_5)] |
| *Chloephaga hybrida* | A | 2.0430 | 0.1415 | 0.7500 | 0.7500 | [[4](#_ENREF_4),[5](#_ENREF_5)] |
| *Chloephaga melanoptera* | A | 3.6400 | 0.1135 | 0.7945 | 0.7945 | [[4](#_ENREF_4),[5](#_ENREF_5)] |
| *Chloephaga picta* | A | 2.6900 | 0.1280 | 0.7808 | 0.7808 | [[4](#_ENREF_4),[5](#_ENREF_5)] |
| *Chloephaga poliocephala* | A | 2.2000 | 0.0971 | 0.4855 | 0.4855 | [[4](#_ENREF_4),[5](#_ENREF_5)] |
| *Chloephaga rubidiceps* | A | 1.4000 | 0.1028 | 0.5140 | 0.5140 | [[4](#_ENREF_4),[5](#_ENREF_5)] |
| *Clangula hyemalis* | A | 0.6360 | 0.0441 | 0.3484 | 0.3484 | [[4](#_ENREF_4),[5](#_ENREF_5)] |
| *Coscoroba coscoroba* | A | 3.8000 | 0.1784 | 1.2131 | 1.2131 | [[4](#_ENREF_4),[5](#_ENREF_5)] |
| *Cyanochen cyanopterus* | A | 1.4200 | 0.0971 | 0.7283 | 0.7283 | [[4](#_ENREF_4),[5](#_ENREF_5)] |
| *Cygnus atratus* | A | 5.1000 | 0.2670 | 1.4685 | 1.4685 | [[4](#_ENREF_4),[5](#_ENREF_5)] |
| *Cygnus bewickii* | A | 5.7000 | 0.2579 | 1.3153 | 1.3153 | [[4](#_ENREF_4),[5](#_ENREF_5)] |
| *Cygnus buccinator* | A | 10.3000 | 0.3665 | 1.9058 | 1.9058 | [[4](#_ENREF_4),[5](#_ENREF_5)] |
| *Cygnus columbianus* | A | 6.2000 | 0.2732 | 1.1748 | 1.1748 | [[4](#_ENREF_4),[5](#_ENREF_5)] |
| *Cygnus cygnus* | A | 8.7500 | 0.3339 | 1.7363 | 1.7363 | [[4](#_ENREF_4),[5](#_ENREF_5)] |
| *Cygnus melanocoryphus* | A | 4.0000 | 0.2474 | 1.1380 | 1.1380 | [[4](#_ENREF_4),[5](#_ENREF_5)] |
| *Cygnus olor* | A | 9.6700 | 0.3530 | 2.6475 | 2.6475 | [[4](#_ENREF_4),[5](#_ENREF_5)] |
| *Dendrocygna arborea* | A | 1.1500 | 0.0488 | 0.4880 | 0.4880 | [[4](#_ENREF_4),[5](#_ENREF_5)] |
| *Dendrocygna arcuata* | A | 0.7320 | 0.0387 | 0.3870 | 0.3870 | [[4](#_ENREF_4),[5](#_ENREF_5)] |
| *Dendrocygna autumnalis* | A | 0.8490 | 0.0443 | 0.5759 | 0.5759 | [[4](#_ENREF_4),[5](#_ENREF_5)] |
| *Dendrocygna bicolor* | A | 0.6900 | 0.0491 | 0.4763 | 0.4763 | [[4](#_ENREF_4),[5](#_ENREF_5)] |
| *Dendrocygna eytoni* | A | 0.7920 | 0.0345 | 0.3795 | 0.3795 | [[4](#_ENREF_4),[5](#_ENREF_5)] |
| *Dendrocygna guttata* | A | 0.8000 | 0.0417 | 0.4587 | 0.4587 | [[4](#_ENREF_4),[5](#_ENREF_5)] |
| *Dendrocygna javanica* | A | 0.5250 | 0.0353 | 0.3530 | 0.3530 | [[4](#_ENREF_4),[5](#_ENREF_5)] |
| *Dendrocygna viduata* | A | 0.6620 | 0.0380 | 0.3990 | 0.3990 | [[4](#_ENREF_4),[5](#_ENREF_5)] |
| *Histrionicus histrionicus* | A | 0.5580 | 0.0544 | 0.3101 | 0.3101 | [[4](#_ENREF_4),[5](#_ENREF_5)] |
| *Hymenolaimus malacorhynchus* | A | 0.7680 | 0.0730 | 0.3942 | 0.3942 | [[4](#_ENREF_4),[5](#_ENREF_5)] |
| *Lophodytes cucullatus* | A | 0.5400 | 0.0576 | 0.5875 | 0.5875 | [[4](#_ENREF_4),[5](#_ENREF_5)] |
| *Lophonetta specularioides* | A | 0.9000 | 0.0569 | 0.3699 | 0.3699 | [[4](#_ENREF_4),[5](#_ENREF_5)] |
| *Malacorhynchus membranaceus* | A | 0.3440 | 0.0352 | 0.2358 | 0.2358 | [[4](#_ENREF_4),[5](#_ENREF_5)] |
| *Marmaronetta angustirostris* | A | 0.4920 | 0.0302 | 0.3564 | 0.3564 | [[4](#_ENREF_4),[5](#_ENREF_5)] |
| *Melanitta fusca* | A | 1.7300 | 0.0920 | 0.7756 | 0.7756 | [[4](#_ENREF_4),[5](#_ENREF_5)] |
| *Melanitta nigra* | A | 0.8000 | 0.0742 | 0.6455 | 0.6455 | [[4](#_ENREF_4),[5](#_ENREF_5)] |
| *Melanitta perspicillata* | A | 0.9000 | 0.0632 | 0.3792 | 0.3792 | [[4](#_ENREF_4),[5](#_ENREF_5)] |
| *Merganetta armata* | A | 0.3270 | 0.0620 | 0.2046 | 0.2046 | [[4](#_ENREF_4),[5](#_ENREF_5)] |
| *Mergus albellus* | A | 0.5680 | 0.0417 | 0.3336 | 0.3336 | [[4](#_ENREF_4),[5](#_ENREF_5)] |
| *Mergus merganser* | A | 1.2320 | 0.0792 | 0.7445 | 0.7445 | [[4](#_ENREF_4),[5](#_ENREF_5)] |
| *Mergus serrator* | A | 0.9080 | 0.0733 | 0.6964 | 0.6964 | [[4](#_ENREF_4),[5](#_ENREF_5)] |
| *Neochen jubata* | A | 1.2500 | 0.0645 | 0.5805 | 0.5805 | [[4](#_ENREF_4),[5](#_ENREF_5)] |
| *Netta erythrophthalma* | A | 0.7660 | 0.0603 | 0.5427 | 0.5427 | [[4](#_ENREF_4),[5](#_ENREF_5)] |
| *Netta peposaca* | A | 1.0040 | 0.0583 | 0.5247 | 0.5247 | [[4](#_ENREF_4),[5](#_ENREF_5)] |
| *Netta rufina* | A | 1.1000 | 0.0568 | 0.5623 | 0.5623 | [[4](#_ENREF_4),[5](#_ENREF_5)] |
| *Nettapus auritus* | A | 0.2600 | 0.0229 | 0.1947 | 0.1947 | [[4](#_ENREF_4),[5](#_ENREF_5)] |
| *Nettapus coromandelianus* | A | 0.2200 | 0.0320 | 0.3200 | 0.3200 | [[4](#_ENREF_4),[5](#_ENREF_5)] |
| *Nettapus pulchellus* | A | 0.3040 | 0.0250 | 0.2500 | 0.2500 | [[4](#_ENREF_4),[5](#_ENREF_5)] |
| *Nomonyx dominicus* | A | 0.3390 | 0.0505 | 0.3030 | 0.3030 | [[4](#_ENREF_4),[5](#_ENREF_5)] |
| *Oxyura australis* | A | 0.8520 | 0.0844 | 0.4642 | 0.4642 | [[4](#_ENREF_4),[5](#_ENREF_5)] |
| *Oxyura jamaicensis* | A | 0.4990 | 0.0713 | 0.5419 | 0.5419 | [[4](#_ENREF_4),[5](#_ENREF_5)] |
| *Oxyura leucocephala* | A | 0.5930 | 0.0970 | 0.5820 | 0.5820 | [[4](#_ENREF_4),[5](#_ENREF_5)] |
| *Oxyura maccoa* | A | 0.5540 | 0.0880 | 0.5280 | 0.5280 | [[4](#_ENREF_4),[5](#_ENREF_5)] |
| *Oxyura vittata* | A | 0.5900 | 0.0787 | 0.3148 | 0.3148 | [[4](#_ENREF_4),[5](#_ENREF_5)] |
| *Plectropterus gambensis* | A | 4.7000 | 0.1388 | 1.3047 | 1.3047 | [[4](#_ENREF_4),[5](#_ENREF_5)] |
| *Polysticta stelleri* | A | 0.8420 | 0.0551 | 0.4408 | 0.4408 | [[4](#_ENREF_4),[5](#_ENREF_5)] |
| *Pteronetta hartlaubi* | A | 0.7880 | 0.0538 | 0.4465 | 0.4465 | [[4](#_ENREF_4),[5](#_ENREF_5)] |
| *Salvadorinia waiguensis* | A | 0.4690 | 0.0570 | 0.1710 | 0.1710 | [[4](#_ENREF_4),[5](#_ENREF_5)] |
| *Sarkidornis melanotos* | A | 1.0690 | 0.0643 | 0.6109 | 0.6109 | [[4](#_ENREF_4),[5](#_ENREF_5)] |
| *Somateria fischeri* | A | 1.3040 | 0.0771 | 0.2853 | 0.2853 | [[4](#_ENREF_4),[5](#_ENREF_5)] |
| *Somateria mollissima* | A | 1.9150 | 0.1110 | 0.4773 | 0.4773 | [[4](#_ENREF_4),[5](#_ENREF_5)] |
| *Somateria spectabilis* | A | 1.5670 | 0.0667 | 0.3335 | 0.3335 | [[4](#_ENREF_4),[5](#_ENREF_5)] |
| *Speculanas specularis* | A | 1.5310 | 0.0690 | 0.3105 | 0.3105 | [[4](#_ENREF_4),[5](#_ENREF_5)] |
| *Stictonetta naevosa* | A | 0.8420 | 0.0772 | 0.5713 | 0.5713 | [[4](#_ENREF_4),[5](#_ENREF_5)] |
| *Tachyeres brachypterus* | A | 3.4500 | 0.1454 | 0.8724 | 0.8724 | [[4](#_ENREF_4),[5](#_ENREF_5)] |
| *Tachyeres leucocephalus* | A | 3.0130 | 0.1324 | 0.6090 | 0.6090 | [[4](#_ENREF_4),[5](#_ENREF_5)] |
| *Tachyeres patachonicus* | A | 2.3460 | 0.1166 | 0.7229 | 0.7229 | [[4](#_ENREF_4),[5](#_ENREF_5)] |
| *Tachyeres pteneres* | A | 4.2280 | 0.1465 | 0.9669 | 0.9669 | [[4](#_ENREF_4),[5](#_ENREF_5)] |
| *Tadorna cana* | A | 1.4170 | 0.0971 | 0.9225 | 0.9225 | [[4](#_ENREF_4),[5](#_ENREF_5)] |
| *Tadorna ferruginea* | A | 1.1000 | 0.0834 | 0.7089 | 0.7089 | [[4](#_ENREF_4),[5](#_ENREF_5)] |
| *Tadorna radjah* | A | 0.8390 | 0.0578 | 0.5202 | 0.5202 | [[4](#_ENREF_4),[5](#_ENREF_5)] |
| *Tadorna tadorna* | A | 1.0430 | 0.0809 | 0.7200 | 0.7200 | [[4](#_ENREF_4),[5](#_ENREF_5)] |
| *Tadorna tadornoides* | A | 1.2910 | 0.0900 | 0.9360 | 0.9360 | [[4](#_ENREF_4),[5](#_ENREF_5)] |
| *Tadorna variegata* | A | 1.3870 | 0.0881 | 0.8281 | 0.8281 | [[4](#_ENREF_4),[5](#_ENREF_5)] |
| *Thalassornis leuconotus* | A | 0.6950 | 0.0826 | 0.6608 | 0.6608 | [[4](#_ENREF_4),[5](#_ENREF_5)] |
| *Casuarius casuarius* | S | 51.0000 | 0.6170 | 3.2907 | 8.2267 | [[3](#_ENREF_3),[6](#_ENREF_6),[7](#_ENREF_7)] |
| *Dromaius novaehollandiae* | S | 35.4000 | 0.5500 | 8.0667 | 12.1000 | [[3](#_ENREF_3),[7](#_ENREF_7)] |
| *Struthio camelus* | S | 91.7500 | 1.5000 | 13.0000 | 19.5000 | [[3](#_ENREF_3),[7](#_ENREF_7),[8](#_ENREF_8)] |
| *Rhea americana* | S | 22.6670 | 0.6000 | 14.2286 | 14.2286 | [[3](#_ENREF_3),[7](#_ENREF_7),[9](#_ENREF_9),[10](#_ENREF_10)] |
| *Pterocnemia pennata* | S | 18.5000 | 0.6230 | 12.7090 | 12.7090 | [[3](#_ENREF_3),[11-14](#_ENREF_11)] |
| *Apteryx australis* | S | 2.8170 | 0.4300 | 0.8600 | 0.8600 | [[3](#_ENREF_3),[7](#_ENREF_7)] |
| *Apteryx owenii* | S | 1.3400 | 0.3010 | 0.4515 | 0.4515 | [[3](#_ENREF_3),[7](#_ENREF_7)] |
| *Alligator mississipiensis* | C | 47.8000 | 0.0766 | 2.7116 | 2.7116 | [[15](#_ENREF_15)] |
| *Alligator sinensis* | C | 14.6000 | 0.0482 | 1.2387 | 1.2387 | [[15](#_ENREF_15)] |
| *Caiman crocodilus* | C | 10.9000 | 0.0629 | 1.5348 | 1.5348 | [[15](#_ENREF_15)] |
| *Caiman latirostris* | C | 14.6000 | 0.0762 | 2.3927 | 2.3927 | [[15](#_ENREF_15)] |
| *Melanosuchus niger* | C | 82.0000 | 0.1436 | 5.6435 | 5.6435 | [[15](#_ENREF_15)] |
| *Paleosuchus palpebrosus* | C | 5.9000 | 0.0686 | 0.9124 | 0.9124 | [[15](#_ENREF_15)] |
| *Paleosuchus trigonatus* | C | 7.5000 | 0.0672 | 1.0147 | 1.0147 | [[15](#_ENREF_15)] |
| *Crocodylus acutus* | C | 76.7000 | 0.1128 | 4.0157 | 4.0157 | [[15](#_ENREF_15)] |
| *Crocodylus cataphractus* | C | 50.5000 | 0.1460 | 2.8470 | 2.8470 | [[15](#_ENREF_15)] |
| *Crocodylus intermedius* | C | 107.9000 | 0.1104 | 4.3939 | 4.3939 | [[15](#_ENREF_15)] |
| *Crocodylus johnsoni* | C | 19.5000 | 0.0697 | 0.8643 | 0.8643 | [[15](#_ENREF_15)] |
| *Crocodylus mindorensis* | C | 36.9000 | 0.0736 | 1.3469 | 1.3469 | [[15](#_ENREF_15)] |
| *Crocodylus moreletii* | C | 31.7000 | 0.0795 | 2.4009 | 2.4009 | [[15](#_ENREF_15)] |
| *Crocodylus niloticus* | C | 94.2000 | 0.1071 | 5.0980 | 5.0980 | [[15](#_ENREF_15)] |
| *Crocodylus novaeguineae* | C | 39.9000 | 0.0885 | 2.6019 | 2.6019 | [[15](#_ENREF_15)] |
| *Crocodylus palustris* | C | 42.7000 | 0.0995 | 2.3283 | 4.6566 | [[15](#_ENREF_15)] |
| *Crocodylus porosus* | C | 78.7000 | 0.1092 | 5.2198 | 5.2198 | [[15](#_ENREF_15)] |
| *Crocodylus rhombifer* | C | 57.5000 | 0.1043 | 2.6492 | 2.6492 | [[15](#_ENREF_15)] |
| *Crocodylus siamensis* | C | 42.5000 | 0.1069 | 3.0360 | 3.0360 | [[15](#_ENREF_15)] |
| *Osteolaemus tetraspis* | C | 18.8000 | 0.0550 | 0.6765 | 0.6765 | [[15](#_ENREF_15)] |
| *Tomistoma schlegelii* | C | 119.0000 | 0.1399 | 4.4488 | 4.4488 | [[15](#_ENREF_15)] |
| *Gavialis gangeticus* | C | 147.0000 | 0.1614 | 6.2785 | 6.2785 | [[15](#_ENREF_15)] |
| *Homopus areolatus* | T | 0.2290 | 0.0090 | 0.0248 | 0.0495 | [[16-20](#_ENREF_16)] |
| *Homopus femoralis* | T | 0.3930 | 0.0070 | 0.0158 | 0.0315 | [[16](#_ENREF_16),[20-23](#_ENREF_20)] |
| *Aldabrachelys gigantea* | T | 93.4000 | 0.0800 | 1.0800 | 2.1600 | [[17](#_ENREF_17),[20](#_ENREF_20),[22](#_ENREF_22),[24](#_ENREF_24)] |
| *Pyxis planicauda* | T | 0.5730 | 0.0175 | 0.0233 | 0.0700 | [[17](#_ENREF_17),[20](#_ENREF_20),[25](#_ENREF_25),[26](#_ENREF_26)] |
| *Astrochelys radiata* | T | 10.2170 | 0.0610 | 0.4889 | 2.6888 | [[20](#_ENREF_20),[22](#_ENREF_22),[27](#_ENREF_27),[28](#_ENREF_28)] |
| *Astrochelys yniphora* | T | 8.0000 | 0.0453 | 0.2082 | 0.8921 | [[17](#_ENREF_17),[20](#_ENREF_20),[22](#_ENREF_22),[29](#_ENREF_29)] |
| *Psammobates tentorius* | T | 0.4870 | 0.0130 | 0.0228 | 0.0796 | [[16](#_ENREF_16),[20](#_ENREF_20),[30](#_ENREF_30)] |
| *Homopus boulengeri* | T | 0.1770 | 0.0110 | 0.0110 | 0.0330 | [[16](#_ENREF_16),[20](#_ENREF_20),[22](#_ENREF_22),[31](#_ENREF_31)] |
| *Chersina angulata* | T | 0.6670 | 0.0275 | 0.0495 | 0.2558 | [[17](#_ENREF_17),[20](#_ENREF_20),[23](#_ENREF_23),[32](#_ENREF_32),[33](#_ENREF_33)] |
| *Chelonoidis carbonaria* | T | 2.0000 | 0.0417 | 0.2381 | 0.8333 | [[17](#_ENREF_17),[20](#_ENREF_20),[34](#_ENREF_34),[35](#_ENREF_35)] |
| *Chelonoidis nigra* | T | 115.1000 | 0.1195 | 1.3657 | 4.5524 | [[20](#_ENREF_20),[22](#_ENREF_22),[24](#_ENREF_24),[36-38](#_ENREF_36)] |
| *Geochelone elegants* | T | 4.8500 | 0.0280 | 0.1319 | 0.3389 | [[17](#_ENREF_17),[20](#_ENREF_20),[22](#_ENREF_22),[39](#_ENREF_39)] |
| *Geochelone sulcata* | T | 43.5330 | 0.0473 | 0.7907 | 2.1745 | [[17](#_ENREF_17),[20](#_ENREF_20),[40](#_ENREF_40),[41](#_ENREF_41)] |
| *Testudo marginata* | T | 1.7560 | 0.0177 | 0.1328 | 0.3186 | [[17](#_ENREF_17),[20](#_ENREF_20),[22](#_ENREF_22),[42](#_ENREF_42),[43](#_ENREF_43)] |
| *Testudo graeca* | T | 2.6120 | 0.0137 | 0.0806 | 0.2580 | [[17](#_ENREF_17),[20](#_ENREF_20),[22](#_ENREF_22),[42-45](#_ENREF_42)] |
| *Testudo horsfieldii* | T | 1.0180 | 0.0175 | 0.0700 | 0.2100 | [[17](#_ENREF_17),[22](#_ENREF_22),[46](#_ENREF_46),[47](#_ENREF_47)] |
| *Testudo hermanni* | T | 1.2490 | 0.0136 | 0.0608 | 0.1013 | [[17](#_ENREF_17),[20](#_ENREF_20),[22](#_ENREF_22),[42](#_ENREF_42),[43](#_ENREF_43),[48](#_ENREF_48),[49](#_ENREF_49)] |
| *Indotestudo elongata* | T | 2.0450 | 0.0470 | 0.1998 | 0.3995 | [[17](#_ENREF_17),[20](#_ENREF_20),[22](#_ENREF_22)] |
| *Manouria emys* | T | 26.0000 | 0.0570 | 2.2990 | 2.2990 | [[20](#_ENREF_20),[22](#_ENREF_22),[50](#_ENREF_50),[51](#_ENREF_51)] |
| *Gopherus agassizii* | T | 1.4000 | 0.0329 | 0.1903 | 0.3807 | [[20](#_ENREF_20),[22](#_ENREF_22),[52](#_ENREF_52),[53](#_ENREF_53)] |

**References**

1. Johnsgard PA (1988) The quails, partridges, and francolins of the world. Oxford: Oxford University Press. 264 p.

2. Lislevand T, Figuerola J, Székely T (2009) Evolution of sexual size dimorphism in grouse and allies (Aves: Phasianidae) in relation to mating competition, fecundity demands and resource division. J Evol Biol 22: 1895-1905.

3. Hoyo Jd, Elliott A, Sargatal J, editors (1992) Handbook of the birds of the world - volume 1 ostrich to ducks. Barcelona: Lynx Edicions. 696 p.

4. Encyclopædia B (2012) anseriform. Encyclopædia Britannica Online. Retrieved 12 November, 2012, from <http://www.britannica.com/EBchecked/topic/26799/anseriform/48962/Reproductive-behaviour>.

5. Figuerola J, Green A (2006) A comparative study of egg mass and clutch size in the Anseriformes. J Ornithol 147: 57-68.

6. Crome FHJ (1976) Some observations on the biology of the cassowary in Northern Queensland. Emu 76: 8-14.

7. Dunning J (1993) CRC handbook of avian body masses. Boca Raton: CRC Press 371 p.

8. Kimwele CN, Graves JA (2003) A molecular genetic analysis of the communal nesting of the ostrich (*Struthio camelus*). Mol Ecol 12: 229-236.

9. Sick H (1985) Ordem Rheiformes. Orintologia Brasileira Uma introducao. Brasilia: Univ. de Brasilia.

10. Dani S (1993) A ema (*Rhea americana*): biologia, manejo e conservacao. Belo Horizonte: Fundacao Acangau. 136 p.

11. Barri FR, Martella MB, Navarro JL (2008) Characteristics, abundance and fertility of orphan eggs of the lesser rhea ( Pterocnemia-Rhea-pennata pennata ): implications for conservation. J Ornithol 149: 285-288.

12. Barri FR, Martella MB, Navarro JL (2009) Reproductive success of wild lesser rheas (*Pterocnemia* -Rhea- *pennata pennata*) in north-western Patagonia, Argentina. J Ornithol 150: 127-132.

13. Navarro JL, Martella MB (2002) Reproductivity and raising of greater rhea (*Rhea americana*) and lesser rhea (*Pterocnemia pennata*) - a review. Arch Gefluegelkd 66: 124-132.

14. Cannon ME, Carpenter RE, Ackerman RA (1986) Synchronous hatching and oxygen consumption of Darwin's rhea eggs (*Pterocnemia pennata*). Physiol Zool 59: 95-108.

15. Thorbjarnarson JB (1996) Reproductive characteristics of the order Crocodylia. Herpetologica 52: 8-24.

16. Hofmeyr MD, Henen BT, Loehr VJT (2005) Overcoming environmental and morphological constraints: egg size and pelvic kinesis in the smallest tortoise, *Homopus signatus*. Can J Zool/Rev Can Zool 83: 1343-1352.

17. Rogner M (1996) Schildkröten 2. Hürtgenwald: Heidi Rogner-Verlag.

18. Branch WR (1989b) *Homopus areolatus*. Parrot-beaked tortoise; Common padloper; Areolated tortoise (English); Padloptjie, Papengaaibekskilpad (Afrikaans). In: Swingland IR, Klemens MW, editors. The conservation biology of tortoises. Gland, Switzerland: Occ. Pap. IUCN/SSC 5, Tortoise and Freshwater Specialists Group. IUCN. pp. 72-74.

19. Loveridge A, Williams EE (1957) Revision of the African tortoises and turtles of the suborder Cryptodira. Bull Mus Comp Zool Harvard 115: 163-557.

20. Bonin F, Devaux B, Dupré A (2006) Turtles of the world. Baltimore: Johns Hopkins Univ. Press. 416 p.

21. Branch WR (1989d) *Homopus femoralis*. Greater padloper, Karoo tortoise (English); Vlakskilpad, Bergskilpadjie, Groter padloper (Afrikaans). In: Swingland IR, Klemens MW, editors. The conservation biology of tortoises. Gland, Switzerland: Occ. Pap. IUCN/SSC 5, Tortoise and Freshwater Specialists Group. IUCN. pp. 80-81.

22. Ernst CH, Barbour RW (2000) Turtles of the world (CD-ROM, Windows Version.1.2). Heidelberg: Springer.

23. Hofmeyr MD (2004) Egg production in *Chersina angulata*: an unusual pattern in a Mediterranean climate. J Herpetol 38: 172-179.

24. Ebersbach K (2001) Zur Biologie und Haltung der Aldabra-Riesenschildkröte (*Geochelone gigantea*) und der Galapagos-Riesenschildkröte (*Geochelone elephantopus*) in menschlicher Obhut unter besonderer Berücksichtigung der Fortpflanzung. Hannover: Dissertation, Tierärztliche Hochschule

25. CITES. Consideration of proposals for amendment of appendices I and II, proposal 55; 2002; Santiago, Chile. Available: <http://www.cites.org/eng/cop/12/prop/index.shtml>. Accessed 2011.

26. Pritchard PCH (1979) Encyclopedia of turtles. Neptune, New Jersey: T. F. H. Publ., Inc. 895 p.

27. Behler JL, Iaderosa. J (1991) A review of the captive breeding program for the radiated tortoise at the New York Zoological Society's Wildlife Survival Center. Proceedings of the first international symposium on turtles and tortoises: conservation and captive husbandry: 160-162.

28. Burchfield PM, Doucette CS, Beimler TF (1980) Captive management of the radiated tortoise *Geochelone radiata* at Gladys Porter Zoo, Brownsville. International Zoo Yearbook 20: 1-6.

29. Pedrono M, Smith LL, Sarovy A (2001) Reproductive ecology of the ploughshare tortoise (*Geochelone yniphora*). J Herpetol 35: 151-156.

30. Leuteritz TEJ, Hofmeyr MD (2007) The extended reproductive season of tent tortoises (*Psammobates tentorius tentorius*): a response to an arid and unpredictable environment. J Arid Environ 68: 546-563.

31. Boycott RC (1989a) *Homopus boulengeri*. Karoo padloper, Boulenger's padloper, Red padloper, Biltong tortoise (English); Karooskilpadjie, Rooiskilpadjie, Donderweerskilpad, Biltongskilpad (Afrikaans). In: Swingland IR, Klemens MW, editors. The conservation biology of tortoises. Gland, Switzerland: Occ. Pap. IUCN/SSC 5, Tortoise and Freshwater Specialists Group. IUCN. pp. 78-79.

32. Mason MC, Kerley GIH, Weatherby CA, Branch WR (2000) Angulate and leopard tortoises in the thicket biome, Eastern Cape, South Africa: populations and biomass estimates. Afr J Ecol 38: 147-153.

33. Vanheezik YM, Cooper J, Seddon PJ (1994) Population characteristics and morphometrics of angulate tortoises on Dassen-Island, South-Africa. J Herpetol 28: 447-453.

34. Rummler G, Rummler MCO (1994) Water-balance of the land tortoise *Geochelone carbonaria* after olfactory bulbectomy. Braz J Med Biol Res 27: 1385-1389.

35. Vanzolini PE (1999) A note on the reproduction of *Geochelone carbonaria* and *G. denticulata* (Testudines, Testudinidae). Rev Bras Biol 59: 593-608.

36. Rostal DC, Robeck TR, Grumbles JS, Burchfield PM, Owens DW (1998) Seasonal reproductive cycle of the Galapagos tortoise (Geochelone nigra) in captivity. Zoo Biol 17: 505-517.

37. Schramm BG, Casares M, Lance VA (1999) Steroid levels and reproductive cycle of the Galapagos tortoise,*Geochelone nigra*, living under seminatural conditions on Santa Cruz Island (Galapagos). Gen Comp Endocrinol 114: 108-120.

38. Zani PA, Gottschall JS, Kram R (2005) Giant Galapagos tortoises walk without inverted pendulum mechanical-energy exchange. J Exp Biol 208: 1489-1494.

39. Vyas R (2005) Captive breeding of the Indian star tortoise (*Geochelone elegans*). Zoo' Print Journal 20: 1859-1864.

40. Grubb P (1971) Comparative notes on the behavior of *Geochelone sulcata* Herpetologica 27: 328-333.

41. Stearns BC (1988) The captive status of the African spurred tortoise *Geochelone sulcata*: recent developments. International Zoo Yearbook 28: 87-98.

42. Hailey A, Loumbourdis NS (1988) Egg size and shape, clutch dynamics, and reproductive effort in European tortoises. Can J Zool/Rev Can Zool 66: 1527-1536.

43. Willemsen RE, Hailey A (2003) Sexual dimorphism of body size and shell shape in European tortoises. J Zool 260: 353-365.

44. Diaz-Paniagua C, Keller C, Andreu AC (1996) Clutch frequency, egg and clutch characteristics, and nesting activity of spur-thighed tortoises, *Testudo graeca*, in southwestern Spain. Can J Zool/Rev Can Zool 74: 560-564.

45. Willemsen RE, Hailey A (2002) Body mass condition in Greek tortoises: regional and interspecific variation. Herpetol J 12: 105-114.

46. Das I (2001) Die Schildkröten des indischen Subkontinents. Frankfurt am Main: Edition Chimaira.

47. Lagarde F, Bonnet X, Henen B, Legrand A, Corbin J, et al. (2003) Sex divergence in space utilisation in the steppe tortoise (*Testudo horsfieldi*). Can J Zool/Rev Can Zool 81: 380-387.

48. Mazzotti S, Pisapia A, Fasola M (2002) Activity and home range of *Testudo hermanni* in Northern Italy. Amphibia-Reptilia 23: 305-312.

49. Willemsen RE, Hailey A (1999) Variation of adult body size of the tortoise *Testudo hermanni* in Greece: proximate and ultimate causes. J Zool 248: 379-396.

50. Abou-Madi N, Jacobson ER (2003) Effects of blood processing techniques on Sodium and Potassium values: a comparison between Aldabra tortoises (Geochelone gigantea) and Burmese Mountain tortoises (Manouria emys). Vet Clin Path 32: 61-66.

51. Louwman JWW (1982) Breeding the six-footed tortoise (*Geochelone emys*) at Wassenaar Zoo. In: Olney PJS, Ellis P, Sommerfelt B, editors. International Zoo Yearbook No 22. London: Zoological Society of London. pp. 153-156.

52. Henen BT (1997) Seasonal and annual energy budgets of female desert tortoises (*Gopherus Agassizii*). Ecology 78: 283-296.

53. Turner FB, Hayden P, Burge BL, Roberson JB (1986) Egg-production by the desert tortoise (*Gopherus agassizii*) in California. Herpetologica 42: 93-104.
