# Supplementary material for: New Insights into Non-Avian Dinosaur Reproduction and Their Evolutionary and Ecological Implications: Linking Fossil Evidence to Allometries of Extant Close Relatives
Source: PLoS One. 2013 Aug 21;8(8):e72862. doi: 10.1371/journal.pone.0072862 (PMC3749170; doi:10.1371/journal.pone.0072862)
Supplement: Table S3 — Results of the OLS regression analyses carried out for body mass against several reproductive traits for tortoises, crocodiles and birds. Allometric functions follow c×BMb (c+b×BM, log-log-plot), where BM is the body mass in kilograms, c is the intercept and b the slope of the line in a log-log-plot. N = sample size. 95% CI = 95% confidence interval of c or b. EM = egg mass. CM = clutch mass. ACM = annual clutch mass. (DOCX) [file pone.0072862.s003.docx]

**Table S3.** Results of the OLS regression analyses carried out for body mass against several reproductive traits for tortoises, crocodiles and birds. Allometric functions follow *c*×BM*^b^* (c+b×BM, log-log-plot), where BM is the body mass in kilograms, *c* is the intercept and *b* the slope of the line in a log-log-plot. N = sample size. 95% CI = 95% confidence interval of *c* or *b*. EM = egg mass. CM = clutch mass. ACM = annual clutch mass.

|  |  | **N** | ***c*** | **95% CI** | | ***b*** | **95% CI** | | **R²** |
| --- | --- | --- | --- | --- | --- | --- | --- | --- | --- |
| **EM vs. BM** | |  |  |  |  |  |  |  |  |
|  | **tortoises** | 20 | 0.019 | [0.015, | 0.023] | 0.355 | [0.256, | 0.454] | 0.759 |
|  | **crocodiles** | 22 | 0.031 | [0.021, | 0.044] | 0.301 | [0.206, | 0.396] | 0.685 |
|  | **birds** | 217 | 0.058 | [0.055, | 0.061] | 0.746 | [0.699, | 0.792] | 0.822 |
| **CM vs. BM** | |  |  |  |  |  |  |  |  |
|  | **tortoises** | 20 | 0.061 | [0.045, | 0.082] | 0.756 | [0.614, | 0.899] | 0.873 |
|  | **crocodiles** | 22 | 0.243 | [0.130, | 0.456] | 0.632 | [0.463, | 0.800] | 0.754 |
|  | **birds** | 217 | 0.420 | [0.398, | 0.444] | 0.709 | [0.656, | 0.763] | 0.758 |
| **ACM vs. BM** | |  |  |  |  |  |  |  |  |
|  | **tortoises** | 20 | 0.165 | [0.121, | 0.227] | 0.742 | [0.593, | 0.890] | 0.860 |
|  | **crocodiles** | 22 | 0.246 | [0.126, | 0.480] | 0.637 | [0.458, | 0.817] | 0.733 |
|  | **birds** | 217 | 0.434 | [0.411, | 0.458] | 0.726 | [0.672, | 0.779] | 0.769 |
