# Supplementary material for: New Insights into Non-Avian Dinosaur Reproduction and Their Evolutionary and Ecological Implications: Linking Fossil Evidence to Allometries of Extant Close Relatives
Source: PLoS One. 2013 Aug 21;8(8):e72862. doi: 10.1371/journal.pone.0072862 (PMC3749170; doi:10.1371/journal.pone.0072862)
Supplement: Table S4 — Summary of results obtained in ANCOVAs. With these analyses we tested for differences in regression slopes ((a); for EM, CM, ACM) and intercepts ((b); CM, ACM) obtained for birds, crocodiles and tortoises under ordinary least squares regression analysis. EM = egg mass, CM = clutch mass, ACM = annual clutch mass, group = categorical variable, coding whether the species is a bird, crocodile or tortoises, log = logarithm to the base 10. n.a. = not applicable. For sample sizes refer to Table S3. (DOCX) [file pone.0072862.s004.docx]

**Table S4.** Summary of results obtained in ANCOVAs. With these analyses we tested for differences in regression slopes ((a); for EM, CM, ACM) and intercepts ((b); CM, ACM) obtained for birds, crocodiles and tortoises under ordinary least squares regression analysis. EM = egg mass, CM = clutch mass, ACM = annual clutch mass, group = categorical variable, coding whether the species is a bird, crocodile or tortoises, log = logarithm to the base 10. n.a. = not applicable. For sample sizes refer to Table S3.

| **Model** | **intercept** | **p-value** | **slope** | **p-value** | **crocodiles** | **p-value** | **tortoises** | **p-value** | **log(BM)**  **:crocodiles** | **p-value** | **log(BM)**  **:tortoises** | **p-value** | **R²** | **p-value** | **df** |
| --- | --- | --- | --- | --- | --- | --- | --- | --- | --- | --- | --- | --- | --- | --- | --- |
| **a)**  **log(EM)~log(BM)*group** | -1.235 | < 2.2e-16 | 0.746 | < 2.2e-16 | -0.279 | 0.0403 | -0.494 | < 2.2e-16 | -0.445 | 6e-07 | -0.390 | 1e-14 | 0.832 | < 2.2e-16 | 253 |
| **log(CM)~log(BM)*group** | -0.376 | < 2.2e-16 | 0.709 | < 2.2e-16 | -0.238 | 0.145 | -0.841 | < 2.2e-16 | -0.078 | 0.457 | 0.047 | 0.415 | 0.852 | < 2.2e-16 | 253 |
| **log(ACM)~log(BM)*group** | -0.363 | < 2.2e-16 | 0.726 | < 2.2e-16 | -0.246 | 0.132 | -0.418 | 3.2e-16 | -0.089 | 0.396 | 0.016 | 0.783 | 0.839 | < 2.2e-16 | 253 |
| **b)** |  |  |  |  |  |  |  |  |  |  |  |  |  |  |  |
| **log(CM)~log(BM)+group** | -0.376 | < 2.2e-16 | 0.716 | < 2.2e-16 | -0.370 | 1.8e-10 | -0.823 | < 2.2e-16 | n.a. | n.a. | n.a. | n.a. | 0.852 | < 2.2e-16 | 255 |
| **log(ACM)~log(BM)+group** | -0.363 | < 2.2e-16 | 0.724 | < 2.2e-16 | -0.383 | 4.7e-11 | -0.411 | < 2.2e-16 | n.a. | n.a. | n.a. | n.a. | 0.838 | < 2.2e-16 | 255 |
