# Supplementary material for: New Insights into Non-Avian Dinosaur Reproduction and Their Evolutionary and Ecological Implications: Linking Fossil Evidence to Allometries of Extant Close Relatives
Source: PLoS One. 2013 Aug 21;8(8):e72862. doi: 10.1371/journal.pone.0072862 (PMC3749170; doi:10.1371/journal.pone.0072862)
Supplement: Table S5 — Summary of results obtained in ANCOVAs. With these analyses we tested for differences in regression slopes ((a); for EM, CM, ACM) and intercepts ((b); CM, ACM) obtained for crocodiles and tortoises under ordinary least squares regression analysis. EM = egg mass, CM = clutch mass, ACM = annual clutch mass, group = categorical variable, coding whether the species is a crocodile or tortoises, log = logarithm to the base 10. n.a. = not applicable. For sample sizes refer to Table S3. (DOCX) [file pone.0072862.s005.docx]

**Table S5.** Summary of results obtained in ANCOVAs. With these analyses we tested for differences in regression slopes ((a); for EM, CM, ACM) and intercepts ((b); CM, ACM) obtained for crocodiles and tortoises under ordinary least squares regression analysis. EM = egg mass, CM = clutch mass, ACM = annual clutch mass, group = categorical variable, coding whether the species is a crocodile or tortoises, log = logarithm to the base 10. n.a. = not applicable. For sample sizes refer to Table S3.

| **Model** | **intercept** | **p-value** | **slope** | **p-value** | **tortoises** | **p-value** | **log(BM)**  **:tortoises** | **p-value** | **R²** | **p-value** | **df** |
| --- | --- | --- | --- | --- | --- | --- | --- | --- | --- | --- | --- |
| **a)**  **log(EM)~log(BM)*group** | -1.514 | 2.07e-15 | 0.301 | 0.00019 | -0.215 | 0.08743 | 0.055 | 0.50577 | 0.882 | < 2.2e-16 | 38 |
| **log(CM)~log(BM)*group** | -0.614 | 0.00142 | 0.632 | 1.32e-06 | -0.603 | 0.00243 | 0.124 | 0.31933 | 0.944 | < 2.2e-16 | 38 |
| **log(ACM)~log(BM)*group** | -0.609 | 0.00236 | 0.637 | 2.57e-06 | -0.172 | 0.38074 | 0.104 | 0.42340 | 0.907 | < 2.2e-16 | 38 |
| **b)** |  |  |  |  |  |  |  |  |  |  |  |
| **log(EM)~log(BM)+group** | -1.583 | < 2.2e-16 | 0.344 | 4.17e-13 | -0.141 | 0.0136 | n.a. | n.a. | 0.880 | < 2.2e-16 | 39 |
| **log(CM)~log(BM)+group** | -0.771 | 1.12e-10 | 0.731 | < 2.2e-16 | -0.436 | 6.32e-06 | n.a. | n.a. | 0.943 | < 2.2e-16 | 39 |
| **log(ACM)~log(BM)+group** | -0.740 | 8.84e-10 | 0.721 | < 2.2e-16 | -0.032 | 0.716 | n.a. | n.a. | 0.905 | < 2.2e-16 | 39 |
