# Supplementary material for: New Insights into Non-Avian Dinosaur Reproduction and Their Evolutionary and Ecological Implications: Linking Fossil Evidence to Allometries of Extant Close Relatives
Source: PLoS One. 2013 Aug 21;8(8):e72862. doi: 10.1371/journal.pone.0072862 (PMC3749170; doi:10.1371/journal.pone.0072862)
Supplement: Table S6 — Allometric models used to estimate reproductive traits of non-avian dinosaurs. EM = egg mass, CM = clutch mass, ACM = annual clutch mass; c = intercept and b = slope of the respective allometry. (DOCX) [file pone.0072862.s006.docx]

**Table S6.** Allometric models used to estimate reproductive traits of non-avian dinosaurs. EM = egg mass, CM = clutch mass, ACM = annual clutch mass; c=intercept and b = slope of the respective allometry.

|  | **Model** | **c** | **95 % CI** | | **b** | **95 % CI** | |
| --- | --- | --- | --- | --- | --- | --- | --- |
| **EM = c*BM^b^** | tortoises | 0.019 | [0.016, | 0.022] | 0.344 | [0.279, | 0.410] |
|  | crocodiles | 0.026 | [0.024, | 0.028] | 0.344 | [0.279, | 0.410] |
|  | birds | 0.058 | [0.055, | 0.061] | 0.746 | [0.699, | 0.792] |
|  |  |  |  |  |  |  |  |
| **CM = c*BM^b^** | tortoises | 0.063 | [0.050, | 0.081] | 0.716 | [0.669, | 0.762] |
|  | crocodiles | 0.180 | [0.156, | 0.207] | 0.716 | [0.669, | 0.762] |
|  | birds | 0.421 | [0.398, | 0.444] | 0.716 | [0.669, | 0.762] |
|  |  |  |  |  |  |  |  |
| **ACM = c*BM^b^** | reptiles | 0.174 | [0.151, | 0.201] | 0.724 | [0.678, | 0.771] |
|  | birds | 0.434 | [0.411, | 0.458] | 0.724 | [0.678, | 0.771] |
